# Mycobacterium tuberculosis Type VII Secretion System Effectors Differentially Impact the ESCRT Endomembrane Damage Response

**DOI:** 10.1128/mBio.01765-18

**Published:** 2018-11-27

**Authors:** Ekansh Mittal, Michael L. Skowyra, Grace Uwase, Emir Tinaztepe, Alka Mehra, Stefan Köster, Phyllis I. Hanson, Jennifer A. Philips

**Affiliations:** a Division of Infectious Diseases, Department of Medicine, Washington University School of Medicine, St. Louis, Missouri, USA; b Department of Molecular Microbiology, Washington University School of Medicine, St. Louis, Missouri, USA; c Department of Cell Biology and Physiology, Washington University School of Medicine, St. Louis, Missouri, USA; d Division of Infectious Diseases, Department of Medicine, New York University School of Medicine, New York, New York, USA; University of Washington

**Keywords:** ESCRT, endomembrane damage, *Mycobacterium tuberculosis*, type VII secretion system, phagosomes

## Abstract

Intracellular pathogens have varied strategies to breach the endolysosomal barrier so that they can deliver effectors to the host cytosol, access nutrients, replicate in the cytoplasm, and avoid degradation in the lysosome. In the case of Mycobacterium tuberculosis, the bacterium perforates the phagosomal membrane shortly after being taken up by macrophages. Phagosomal damage depends upon the mycobacterial ESX-1 type VII secretion system (T7SS). Sterile insults, such as silica crystals or membranolytic peptides, can also disrupt phagosomal and endolysosomal membranes. Recent work revealed that the host endosomal sorting complex required for transport (ESCRT) machinery rapidly responds to sterile endolysosomal damage and promotes membrane repair. We hypothesized that ESCRTs might also respond to pathogen-induced phagosomal damage and that M. tuberculosis could impair this host response. Indeed, we found that ESCRT-III proteins were recruited to M. tuberculosis phagosomes in an *ESX-1*-dependent manner. We previously demonstrated that the mycobacterial effectors EsxG/TB9.8 and EsxH/TB10.4, both secreted by the ESX-3 T7SS, can inhibit ESCRT-dependent trafficking of receptors to the lysosome. Here, we additionally show that ESCRT-III recruitment to sites of endolysosomal damage is antagonized by EsxG and EsxH, both within the context of M. tuberculosis infection and sterile injury. Moreover, EsxG and EsxH themselves respond within minutes to membrane damage in a manner that is independent of calcium and ESCRT-III recruitment. Thus, our study reveals that T7SS effectors and ESCRT participate in a series of measures and countermeasures for control of phagosome integrity.

## Abstract (precis)

Mycobacterium tuberculosis causes tuberculosis, which kills more people than any other infection. M. tuberculosis grows in macrophages, cells that specialize in engulfing and degrading microorganisms. Like many intracellular pathogens, in order to cause disease, M. tuberculosis damages the membrane-bound compartment (phagosome) in which it is enclosed after macrophage uptake. Recent work showed that when chemicals damage this type of intracellular compartment, cells rapidly detect and repair the damage, using machinery called the endosomal sorting complex required for transport (ESCRT). Therefore, we hypothesized that ESCRT might also respond to pathogen-induced damage. At the same time, our previous work showed that the EsxG-EsxH heterodimer of M. tuberculosis can inhibit ESCRT, raising the possibility that M. tuberculosis impairs this host response. Here, we show that ESCRT is recruited to damaged M. tuberculosis phagosomes and that EsxG-EsxH undermines ESCRT-mediated endomembrane repair. Thus, our studies demonstrate a battle between host and pathogen over endomembrane integrity.

## INTRODUCTION

Uptake and lysosomal degradation of microorganisms are a central feature of innate immunity. In response, pathogens have diverse strategies to prevent their destruction in the host endolysosomal system. Viruses and certain bacterial pathogens, such as Listeria and Shigella, escape from the endolysosomal system and replicate in the cytosol. Bacteria that replicate in a membranous vacuole, such as Legionella and Salmonella, also have to gain access to the host cytosol. They rely upon specialized secretion systems to release factors that breach the phagosomal membrane and deliver effectors to the host cytoplasm that reroute cellular trafficking to generate a replicative compartment for the bacilli. In the case of Mycobacterium tuberculosis, the ESX-1 type VII secretion system (T7SS) is essential for bacterial access to the host cytosol (1–4). Host mechanisms that sense and potentially repair microorganism-induced endolysosomal damage are not well understood.

Recent work demonstrated that the endosomal sorting complex required for transport (ESCRT) machinery is rapidly recruited to acutely injured endolysosomes (5), raising the possibility that ESCRTs might also respond to pathogen-induced damage. ESCRT machinery promotes budding and fission of membranes in diverse contexts, including the formation of multivesicular endosomes, cytokinetic abscission, plasma membrane repair, and viral budding (6, 7). ESCRTs consist of four complexes: ESCRT-0, -I, -II, and -III. Upstream ESCRT complexes and a variety of adaptors direct the assembly of ESCRT-III at sites of action, where it performs a membrane-remodeling function. CHMP1 to -7 and IST1, which are monomeric in the cytoplasm, form polymeric ESCRT-III filaments that drive membrane budding or scission. Our finding that ESCRT-III promotes repair of small perforations in the endolysosomal membrane (5) led us to investigate whether macrophages employ ESCRT machinery in the context of pathogen-induced damage and whether M. tuberculosis manipulates this host response.

We reasoned that the response of the ESCRT system to M. tuberculosis would depend upon the mycobacterial ESX-1 T7SS. Numerous studies have demonstrated that both M. tuberculosis and Mycobacterium marinum Δ*esxA* mutants, which lack the ESX-1 secreted effector EsxA/ESAT-6, do not perforate the phagosome, and they have less membrane lytic activity than wild-type (WT) mycobacteria (2–4, 8–12). Because the ESX-1 secretion system as a whole is inactive in Δ*esxA* mutants, the mechanism of phagosomal damage has not been clearly defined but likely requires at least one additional factor that is cosecreted with EsxA by the ESX-1 T7SS (10, 12–17). Recent work shows that the mycobacterial lipid phthicerol dimycocerosate (PDIM) also works in concert with the ESX-1-dependent activity to mediate phagosomal damage (18–20). Damage to the M. tuberculosis phagosome is central to the pathogens’ success, and Δ*esxA* and PDIM mutants do not grow well in macrophages or cause disease in mice (21–24). Presumably the ability of M. tuberculosis to perforate the phagosome is critical to virulence because that is how M. tuberculosis delivers effectors to the cytosol (25). Phagosomal damage may also provide the bacilli access to important nutrients. ESX-1-dependent membrane damage happens early after bacterial uptake, as host sensors present in the cytosol detect bacterial products and respond within the first hours of infection (8, 26–32). During the course of infection, there is increased phagosomal damage, and some bacilli eventually translocate to the cytosol (2, 4, 8, 33).

Our previous studies demonstrated both the importance of ESCRTs in microbial control and that the mycobacterial effectors EsxG and EsxH can antagonize ESCRT-dependent functions. ESCRT restricts the growth of M. tuberculosis in macrophages, demonstrating that this machinery plays an intrinsic role in immunity (34–36). The M. tuberculosis-secreted protein EsxH/TB10.4 can inhibit ESCRT-dependent receptor trafficking (35). EsxH is secreted as a heterodimer with EsxG/TB9.8 by the ESX-3 T7SS. ESX-3 is one of five T7SSs in M. tuberculosis and was initially shown to play a role in metal homeostasis for the bacilli (37–40). In addition to *esxG* and *esxH*, the ESX-3 locus contains *pe5* and *ppe4*, members of the proline-glutamic acid (PE) and proline-proline-glutamic acid (PPE) families, respectively. Δ*esxH* mutants fail to secrete EsxG, EsxH, and PE5, whereas the Δ*pe5-ppe4* mutant still secretes EsxG-EsxH (41). Importantly, by analyzing both Δ*esxH* and Δ*pe5-ppe4* mutants, we were able to separate phenotypes related to metal homeostasis from those attributable to EsxG-EsxH, because although they both have the same iron phenotype, the Δ*pe5-ppe4* mutant still secretes EsxG-EsxH (41). Thus, comparison of these strains allowed us to show that EsxH plays an iron-independent role in virulence, as the Δ*esxH* mutant is remarkably attenuated *in vivo*, whereas the Δ*pe5-ppe4* mutant strain is not (41). EsxG-EsxH from M. tuberculosis, but not the nonpathogen Mycobacterium smegmatis, targets the ESCRT-0 protein, hepatocyte growth factor-regulated tyrosine kinase substrate (HGS/HRS) (35). The EsxG-EsxH heterodimer directly binds HRS, and ectopic expression of EsxG-EsxH inhibits the ability of ESCRT to traffic receptors to the lysosome (35). In addition, EsxG-EsxH impairs major histocompatibility complex class II (MHC-II) antigen presentation, which appears related to its ability to antagonize ESCRT (42). Thus, we hypothesized that EsxG-EsxH might impair ESCRT’s response to endolysosomal damage.

M. tuberculosis that breaches the phagosomal system activates autophagy, which sequesters targeted material in a double-membrane LC3-marked compartment that is delivered to an autophagolysosome for degradation (3, 43, 44). Similarly, damaged lysosomes are engulfed by autophagosomes so they can be cleared in a process referred to as lysophagy (45–48). The rapid response of ESCRTs to endolysosomal damage has distinct features from lysophagy (5). ESCRTs respond to small perturbations in the membrane, which are permeable to protons and small molecules, whereas larger membrane disruptions appear to be required to activate lysophagy. ESCRTs respond to damage more rapidly than factors directly linked to autophagy, and their recruitment depends upon the ESCRT-nucleating factors TSG101 and ALIX but not upon the autophagy protein ATG16L1 (5). While autophagy has been extensively studied in the context of M. tuberculosis and other intracellular pathogens (49–51), whether there is also an early and independent host response to phagosomal perforation has not been explored. Here, we show that ESCRT machinery is recruited to M. tuberculosis phagosomes in an *ESX-1*-dependent manner, while at the same time EsxG-EsxH antagonizes this host ESCRT response. These studies reveal the interplay of T7SS effectors and ESCRT in the host response to endomembrane damage during M. tuberculosis infection.

## RESULTS

### ESCRT-III is recruited to *M. tuberculosis* phagosomes in an *ESX-1*-dependent manner.

**FIG 1:**
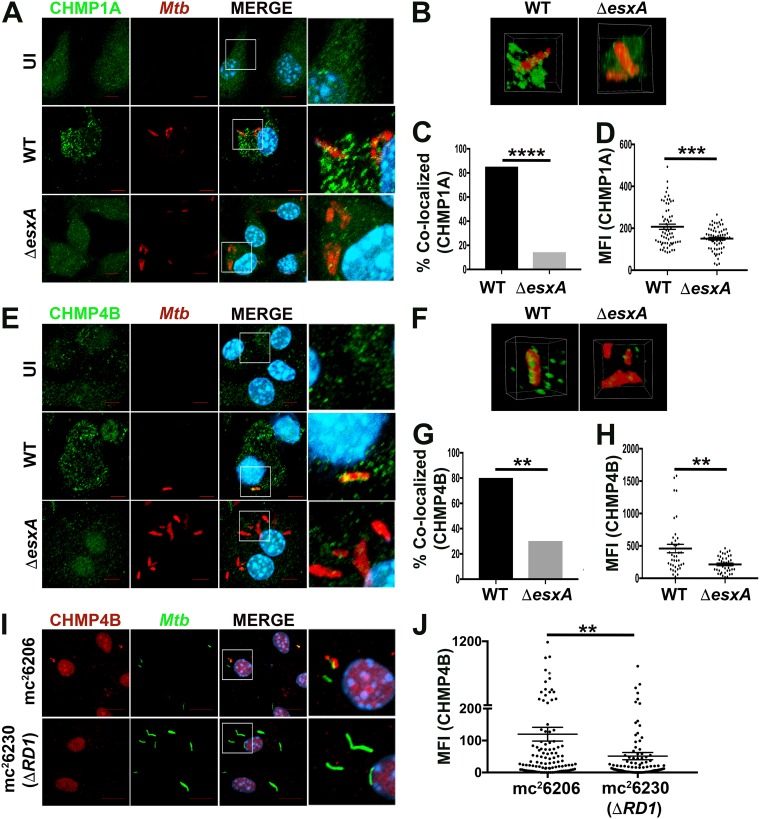
ESCRT-III is recruited to M. tuberculosis (*Mtb*) phagosomes in an *esxA*-dependent manner. (A, E, and I) Immunofluorescence (IF) images of CHMP1A (A) and CHMP4B (E and I) in BMDMs that were uninfected (UI) or infected with PKH-labeled H37Rv (WT) or the Δ*esxA* mutant or GFP-expressing mc^2^6206 or mc^2^6230 (Δ*RD1*) for 3 h. Images are maximum-intensity projections. Scale bar, 10 μm. Boxed areas in the merged image are shown in higher magnification in the rightmost panel. (B and F) Three-dimensional renderings of individual bacilli, which are also shown in Movies S1 to S4. (C and G) The percentages of bacteria in CHMP1A- and CHMP4B-positive phagosomes were quantified from over 100 bacteria by an individual blind to sample identity (****, *P* ≤ 0.0001 for CHMP1A, and **, *P* ≤ 0.0079 for CHMP4B, Fisher's exact test). Automated image analysis was used to quantify the mean fluorescence intensity (MFI) of CHMP1A (D) and CHMP4B (H and J) colocalized with individual bacilli from 5 fields from a 12-mm coverslip. Data are means ± SEM from one representative experiment of three for H37Rv strains or two independent experiments for mc^2^6206 and mc^2^6230. *, *P* ≤ 0.05, **, *P* ≤ 0.01, and ***, *P* ≤ 0.001, Student's *t* test.

ESCRT-III is recruited to endolysosomes after damage with the lysosomotropic compound LLOME (l-leucyl–l-leucine *O*-methyl ester) in a variety of cell types, including phagocytic THP-1 cells (5). We verified that the ESCRT-III proteins CHMP1A, CHMP1B, and CHMP4B rapidly respond to LLOME-induced damage in bone marrow-derived macrophages (BMDMs) (see Fig. S1 in the supplemental material). To determine whether ESCRT-III is also recruited to perforated M. tuberculosis phagosomes, we compared CHMP1A localization in wild-type (WT) M. tuberculosis strain H37Rv- and Δ*esxA* mutant*-*infected BMDMs. We used a Δ*esxA* mutant strain that has been extensively characterized and is defective in damaging the phagosome (32, 52, 53). We examined cells 3 h postinfection, before WT bacilli exhibit extensive phagosomal disruption or overt translocation to the cytosol (2, 4, 32). After infection with WT M. tuberculosis, 85.2% of bacilli were associated with CHMP1A staining, which appeared in a punctate pattern adjacent to the bacilli (Fig. 1A to C; see Movie S1 in the supplemental material). In contrast, only 14.3% of the Δ*esxA* mutants were colocalized with CHMP1A (Fig. 1A to C; see Movie S2 in the supplemental material). In addition, in the cases where Δ*esxA* mutant bacilli were associated with CHMP1A, the staining was less extensive compared to that seen with WT (Fig. 1A and B). The difference in the degree of CHMP1A colocalization was apparent when we quantified the phagosomal CHMP1A signal using automated image analysis as previously described (35, 52, 54) (Fig. 1D). As for CHMP1A, CHMP4B was also more extensively colocalized with WT M. tuberculosis than the Δ*esxA* mutant (Fig. 1E to H; see Movies S3 and S4 in the supplemental material). To verify the role of *ESX-1*, we compared two candidate vaccine strains of M. tuberculosis: mc^2^6230, which carries a deletion (Δ*RD1*) that is similar to the one disrupting *ESX-1* in Mycobacterium bovis BCG, and mc^2^6206, in which *ESX-1* is intact. We found reduced CHMP4B recruitment to the Δ*RD1* strain, as we had for the Δ*esxA* mutant (Fig. 1I and J). Overall, we conclude that ESCRT-III is recruited to M. tuberculosis phagosomes in an *ESX-1*-dependent manner, consistent with its recruitment to sites of endomembrane damage (5). Interestingly, in addition to recruitment to M. tuberculosis phagosomes, there was an overall change in the cellular distribution of CHMP1A and CHMP4B in macrophages infected with M. tuberculosis compared to uninfected cells. In uninfected macrophages, ESCRT-III proteins appeared diffusely cytosolic; M. tuberculosis infection resulted in a punctate distribution, similar to that seen in cells treated with lysosomolytic LLOME (5). This punctate distribution was less pronounced during infection with the Δ*esxA* mutant, revealing that M. tuberculosis, in an *ESX-1*-dependent manner, causes widespread endomembrane damage or otherwise manipulates ESCRT-III localization.

10.1128/mBio.01765-18.1 FIG S1 ESCRT-III is recruited to damaged endolysosomes in macrophages. BMDMs were treated with 1 mM LLOME or solvent control for 15 min. Immunofluorescence microscopy shows CHMP1A (A), CHMP1B (B), and CHMP4B (C) in green. Images are maximum-intensity projections. Nuclei were stained with DAPI. Scale bar, 10 μm. Download FIG S1, JPG file, 1.9 MB. Copyright © 2018 Mittal et al. 2018 Mittal et al. This content is distributed under the terms of the Creative Commons Attribution 4.0 International license.

10.1128/mBio.01765-18.7 MOVIE S1 CHMP1A in WT M. tuberculosis-infected cells. The movie shows the three-dimensional immunofluorescence image of CHMP1A in BMDMs infected with WT M. tuberculosis, as shown in the left panel of Fig. 1B. Download Movie S1, MOV file, 3.2 MB. Copyright © 2018 Mittal et al. 2018 Mittal et al. This content is distributed under the terms of the Creative Commons Attribution 4.0 International license.

10.1128/mBio.01765-18.8 MOVIE S2 CHMP1A in Δ*esxA* mutant-infected cells. The movie shows the three-dimensional immunofluorescence image of CHMP1A in BMDMs infected with the Δ*esxA* mutant, as shown in the right panel of Fig. 1B. Download Movie S2, MOV file, 3.7 MB. Copyright © 2018 Mittal et al. 2018 Mittal et al. This content is distributed under the terms of the Creative Commons Attribution 4.0 International license.

10.1128/mBio.01765-18.9 MOVIE S3 CHMP4B in WT M. tuberculosis-infected cells. The movie shows the three-dimensional immunofluorescence image of CHMP4B in BMDMs infected with WT M. tuberculosis, as shown in the left panel of Fig. 1F. Download Movie S3, MOV file, 2.3 MB. Copyright © 2018 Mittal et al. 2018 Mittal et al. This content is distributed under the terms of the Creative Commons Attribution 4.0 International license.

10.1128/mBio.01765-18.10 MOVIE S4 CHMP4B in Δ*esxA* mutant-infected cells. The movie shows the three-dimensional immunofluorescence image of CHMP4B in BMDMs infected with the Δ*esxA* mutant, as shown in the right panel of Fig. 1F. Download Movie S4, MOV file, 2.6 MB. Copyright © 2018 Mittal et al. 2018 Mittal et al. This content is distributed under the terms of the Creative Commons Attribution 4.0 International license.

### EsxG-EsxH inhibits ESCRT-III recruitment to phagosomes.

**FIG 2:**
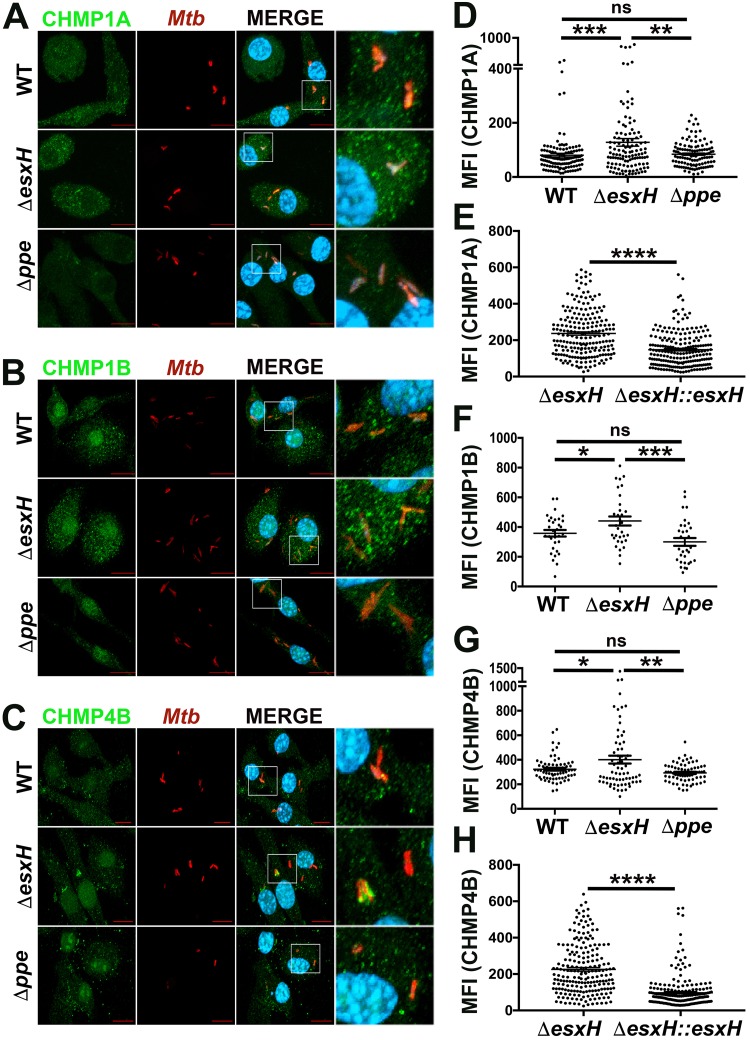
EsxH antagonizes ESCRT-III recruitment. (A, B, and C) IF images of CHMP1A (A), CHMP1B (B), and CHMP4B (C) with DsRed-expressing H37Rv or Δ*esxH* and Δ*pe5-ppe4* (Δ*ppe*) mutants 3 hpi in BMDMs. Images are maximum-intensity projections. Scale bar, 10 μm. Boxed areas in the merged image are shown in higher magnification in the rightmost panel. *Mtb*, M. tuberculosis. (D to H) Automated image analysis was used to quantify the MFI of CHMP1A (D and E), CHMP1B (F), and CHMP4B (G and H) colocalized with individual bacilli in 5 fields from a 12-mm coverslip. In panels E and H, BMDMs were infected with the PKH-labeled Δ*esxH* or Δ*esxH*::*esxH* mutant for 3 h. Data are means ± SEM from one representative experiment from three (for the WT and Δ*esxH* mutant in panels A to D, F, and G and the Δ*pe5-ppe4* mutant in panels A, C, D, and G) or two (for panels E and H and the Δ*pe5-ppe4* mutant in panels B and F) independent experiments. *, *P* ≤ 0.05, **, *P* ≤ 0.01, ***, *P* ≤ 0.001, and ****, *P* ≤ 0.0001, Student’s *t* test. ns, not significant.

Next we asked whether EsxG-EsxH influences ESCRT-III recruitment to phagosomes by comparing the association of CHMP1A, CHMP1B, and CHMP4B with WT and Δ*esxH* mutant bacilli. In contrast to our findings with the Δ*esxA* mutant, which showed reduced ESCRT-III recruitment, we found more robust colocalization of CHMP1A, CHMP1B, and CHMP4B with the Δ*esxH* mutants (Fig. 2A to H). ESCRT-III recruitment to the Δ*esxH* mutant was reduced toward WT levels when EsxG-EsxH was provided to the Δ*esxH* mutant on an integrating plasmid (Fig. 2E and H). Macrophages that are infected with a high intracellular burden of M. tuberculosis undergo host cell death in a manner that depends upon lysosomal proteases, suggesting that under conditions of high M. tuberculosis burden, endolysosomal damage is more extensive (55). Interestingly, the recruitment of ESCRT-III to a subset of Δ*esxH* bacilli was particularly prominent in heavily infected macrophages (see Fig. S2A and B in the supplemental material). The increase in ESCRT-III recruitment during infection did not reflect a change in the amount of CHMP1A or CHMP1B protein, which was unaffected by infection or the presence of *esxH,* even at multiplicities of infection (MOI) up to 50 (Fig. S2C).

10.1128/mBio.01765-18.2 FIG S2 ESCRT-III recruitment to WT and Δ*esxH* phagosomes in heavily infected macrophages. Shown are IF images of CHMP1A (A) and CHMP1B (B) in BMDMs that were heavily infected with the DsRED-expressing H37Rv (WT) and Δ*esxH* mutant for 3 h. Images are maximum-intensity projections. Scale bar, 10 μm. *Mtb*, M. tuberculosis. (C) BMDMs were uninfected or infected with the WT or Δ*esxH* mutant for 2 to 4 h at a multiplicity of infection (MOI) of 10 to 50 as indicated. CHMP1A, CHMP1B, and β-actin were examined by Western blotting. Download FIG S2, JPG file, 2.5 MB. Copyright © 2018 Mittal et al. 2018 Mittal et al. This content is distributed under the terms of the Creative Commons Attribution 4.0 International license.

To determine whether the enhanced ESCRT recruitment is related to the defect in iron acquisition by the Δ*esxH* mutant, we compared the Δ*esxH* mutant to the Δ*pe5-ppe4* mutant. The ESX-3 machinery secretes EsxG, EsxH, and PE5 (41). Δ*esxH* and Δ*pe5-ppe4* mutants are defective in growing on solid medium, unless the medium is supplemented with exogenous iron (41). However, while the Δ*esxH* mutant fails to secrete EsxG, EsxH, and PE5, the Δ*pe5-ppe4* mutant still secretes EsxG-EsxH (41). Since secretion of EsxG-EsxH is absent in the Δ*esxH* strain and present in the Δ*pe5-ppe4* mutant, by analyzing both mutants, we can separate phenotypes related to metal homeostasis, impaired in both, to phenotypes attributable to EsxG-EsxH, impaired exclusively in the Δ*esxH* mutant. When we examined phagosomal recruitment of CHMP1A, CHMP1B, or CHMP4B to Δ*pe5-ppe4* phagosomes, there was no difference compared to infection with WT M. tuberculosis (Fig. 2A to D, F, and G). We conclude that the effect of *esxH* on ESCRT-III is not related to its role in iron metabolism. Thus, whereas ESX-1 promotes ESCRT-III recruitment to M. tuberculosis phagosomes (Fig. 1), EsxG-EsxH impairs recruitment.

### EsxG-EsxH alters the phagolysosomal damage response and trafficking.

**FIG 3:**
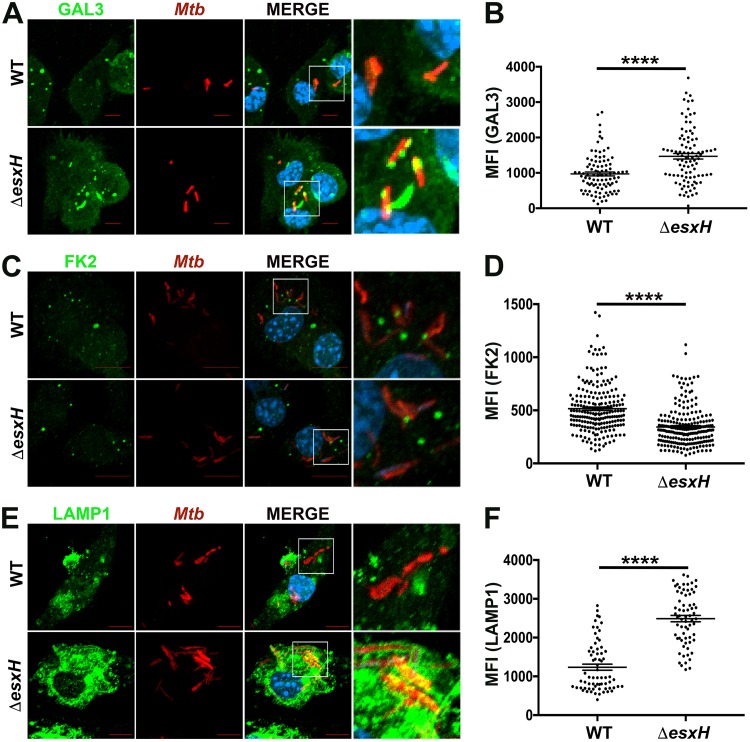
EsxG-EsxH alters phagosomal GAL3, ubiquitin, and LAMP1. (A, C, and E) IF images of GAL3 (A), ubiquitin (FK2 antibody) (C), and LAMP1 (E) in BMDMs that were infected with DsRed-expressing H37Rv (WT) or the Δ*esxH* mutant for 3 h. Images are maximum-intensity projections. Scale bar, 10 μm. Boxed areas in the merged image are shown in higher magnification in the rightmost panel. *Mtb*, M. tuberculosis. (B, D, and F) Automated image analysis was used to quantify the MFI of GAL3 (B), ubiquitin (D), and LAMP1 (F) colocalized with individual bacilli from 5 fields of a 12-mm coverslip. Data are means ± SEM from one representative experiment from three (A, B, E, and F) or two (C and D) independent experiments. ****, *P* ≤ 0.0001, Student's *t* test.

Extensively damaged phagosomes and endosomes can be detected by cytoplasmic galectins, such as GAL3, which recognize disrupted compartments by binding to lumenal glycans that become exposed to the cytosol (56). In addition, phagosomal damage triggers protein ubiquitination. Both ubiquitinated proteins and galectins recruit autophagy receptors, such as p62, CALCOCO2/NDP52, and NBR1 to phagosomes disrupted by mycobacteria (3, 52, 56–58). Accordingly, GAL3 and ubiquitin are both reduced on phagosomes containing the Δ*esxA* mutant, which does not efficiently damage the phagosome (3, 32, 52). We next asked whether the Δ*esxH* mutant colocalized more with GAL3 and conjugated ubiquitin, as it did with ESCRT-III. Interestingly, phagosomes containing Δ*esxH* mutants exhibited increased colocalization with GAL3 compared to phagosomes containing WT bacilli, whereas ubiquitin association was reduced (Fig. 3A to D). Hence, the Δ*esxH* mutant appears to provoke an enhanced and altered response to damage.

To determine whether the altered response to phagosomal damage influenced phagosomal maturation, we examined the colocalization of WT, Δ*esxH*, and Δ*pe5-ppe4* bacteria with LAMP1, a late endosomal and lysosomal marker. There was markedly enhanced colocalization between LAMP1 and the Δ*esxH* mutant compared to WT bacilli (Fig. 3E and F). The enhanced LAMP1 localization with the Δ*esxH* mutant was complemented by EsxG-EsxH expressed from an integrated plasmid (see Fig. S3A and B in the supplemental material). In the case of the Δ*pe5-ppe4* mutant, there was a statistically significant, but very modest, difference in LAMP1 colocalization based upon automated quantification, which was subtle upon visual inspection (Fig. S3C and D). We conclude that in addition to impairing recruitment of ESCRTs to mycobacterium-containing phagosomes, EsxG-EsxH also impedes the conversion of phagosomes into lysosomes, consistent with our previous studies showing that M. tuberculosis strains engineered to overexpress EsxG-EsxH are better able to arrest phagosome maturation than WT strains (35).

10.1128/mBio.01765-18.3 FIG S3 Lysosomal trafficking of the Δ*pe5-ppe4* mutant and Δ*esxH::esxH* complemented strain. (A and C) IF images of LAMP1 in BMDMs infected with (A) mCherry-expressing Δ*esxH* and Δ*esxH*::*esxH* strains or (C) the DsRed-expressing H37Rv (WT) and Δ*pe5-ppe4* **(**Δ*ppe*) mutant 3 hpi. Images are maximum-intensity projections. Scale bar, 10 μm. *Mtb*, M. tuberculosis. (B and D) Automated image analysis was used to quantify the MFI of LAMP1 colocalized with individual bacilli in 5 different fields. Data are means ± SEM from one representative experiment from at least two independent experiments. *, *P* ≤ 0.05; ****, *P* ≤ 0.0001. Download FIG S3, JPG file, 2.0 MB. Copyright © 2018 Mittal et al. 2018 Mittal et al. This content is distributed under the terms of the Creative Commons Attribution 4.0 International license.

### EsxG-EsxH impairs ESCRT-III recruitment to damaged endomembranes.

**FIG 4:**
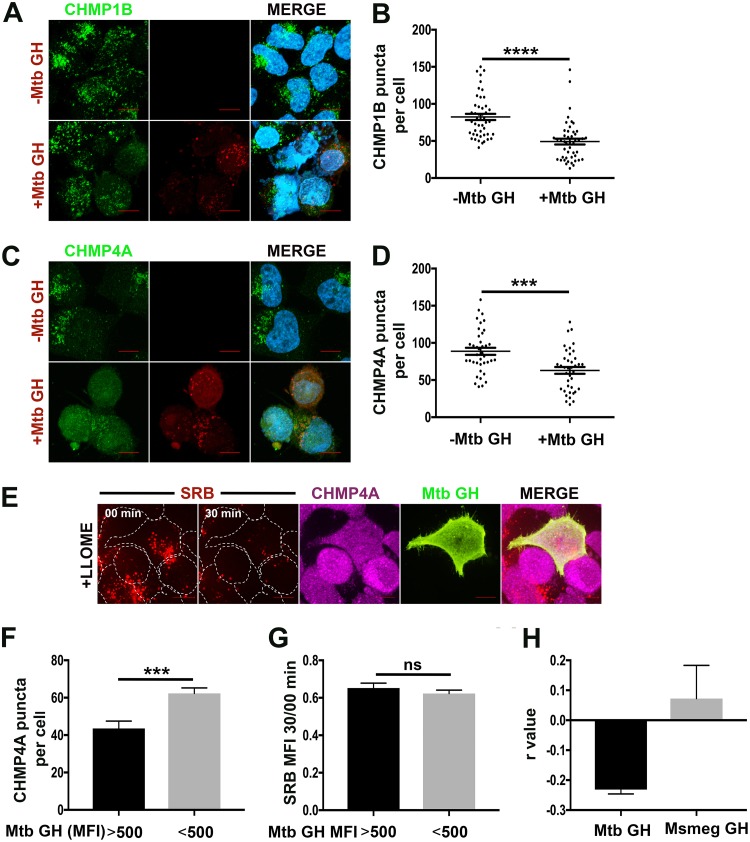
EsxG-EsxH impairs ESCRT-III recruitment to damaged lysosomes. (A and C) HeLa cells transfected with M. tuberculosis (Mtb) EsxG-EsxH (GH) or the vector control were treated with LLOME or the solvent control and stained for CHMP1B (A) or CHMP4A (C). ESCRT-III and EsxG-EsxH are shown in green and red, respectively. EsxG-EsxH was visualized with an anti-EsxG-EsxH monoclonal antibody. (B and D) Automated image analysis was used to quantify the number of CHMP1B (A) or CHMP4A (B) punctae on 30 macrophages per sample. Data are means ± SEM from one representative experiment from at least three independent experiments. ***, *P* ≤ 0.001, and ****, *P* ≤ 0.0001, Student's *t* test. (E) HeLa cells were transfected with Mtb EsxG-EsxH and loaded with SRB. Live-cell imaging was used to visualize SRB before and after addition of LLOME, after which cells were fixed and stained to visualize EsxG-EsxH (green) and CHMP4A (magenta). Image panels of representative cells are shown at the times indicated from each recording. Individual cells are outlined by white dashed lines. Images are maximum-intensity projections. Scale bars, 10 µm. (F and G) Automated image analysis was used to quantify the number of CHMP4A punctae, the reduction in SRB signal, and the MFI of EsxG-EsxH on a per cell basis. The number of CHMP4A punctae and the reduction is SRB signal were compared in cells with an EsxG-EsxH MFI greater than and less than 500. Data are means ± SEM from one representative experiment from at least three independent experiments. ***, *P* ≤ 0.001, Student’s *t* test. ns, not significant. (H) HeLa cells transfected with Mtb or M. smegmatis (Msmeg) EsxG-EsxH were treated with LLOME, and CHMP4A and EsxG-EsxH were visualized. Automated image analysis was used to quantify the EsxG-EsxH MFI and the number of CHMP4A punctae in individual cells. The correlation between EsxG-EsxH expression and number of CHMP4A punctae is shown (*R* value). Data are means ± SEM from four independent experiments in which at least 100 cells were evaluated.

Given the complexity of the host-pathogen interactions during infection, we turned to a simplified system to clarify the apparent antagonism between EsxG-EsxH and damage-triggered ESCRT recruitment. We compared the ESCRT response to LLOME in HeLa cells transfected with a plasmid encoding EsxG-EsxH to cells transfected with a control vector. LLOME treatment for 15 min caused robust formation of ALIX, CHMP1B, CHMP4A, and IST1 punctae, as previously reported (see Fig. S4 in the supplemental material) (5). Interestingly, cells transfected with EsxG-EsxH had fewer CHMP1B and CHMP4A punctae than vector control cells (Fig. 4A to D). The reduction in CHMP1B and CHMP4A punctae was not caused by less ESCRT-III protein, as EsxG-EsxH did not impact their protein levels based upon Western blotting (see Fig. S5 in the supplemental material). We considered the possibility that M. tuberculosis EsxG-EsxH might reduce LLOME-induced damage, rather than impairing the ESCRT-III response to such damage. To assess the degree of membrane damage induced by LLOME, we preloaded the endolysosomal system with sulforhodamine B (SRB), a 559-Da fluorescent, membrane-impermeable molecule. Using live-cell imaging, we observed that the majority of SRB punctae were gone within 30 min of LLOME treatment, reflecting a loss of endomembrane integrity (Fig. 4E). In cells transiently transfected with EsxG-EsxH, we assessed the loss of SRB fluorescence and appearance of CHMP4A punctae on an individual cell basis. We found that expression of M. tuberculosis EsxG-EsxH was negatively correlated with the number of CHMP4A punctae; however, there was no correlation between EsxG-EsxH expression and loss of SRB signal (Fig. 4E to G). When we compared those cells with in which the EsxG-EsxH MFI exceeded an empirical threshold to those with a lower mean fluorescent intensity (MFI), cells with the brighter EsxG-EsxH signal had fewer CHMP4A punctae, but there was no difference in the response of SRB (Fig. 4E to G). This demonstrates that EsxG-EsxH did not impair the ability of LLOME to cause membrane damage, but rather impaired the response to damage. In addition, we previously showed that M. tuberculosis EsxG-EsxH can inhibit ESCRT-dependent trafficking functions in cells, whereas EsxG-EsxH from the nonpathogenic species M. smegmatis does not (35). Consistent with that observation, only M. tuberculosis EsxG-EsxH was negatively correlated with the number of CHMP4A punctae (Fig. 4H). Overall, we conclude that M. tuberculosis EsxG-EsxH can impair the recruitment of ESCRT-III to endomembrane damage.

10.1128/mBio.01765-18.4 FIG S4 ESCRT-III is recruited to damaged endolysosomes in HeLa cells. HeLa cells were treated with 1 mM LLOME or the solvent control for 15 min. IF shows ALIX (A), CHMP1B (B), CHMP4A (C), and IST1 (D) in green. Images are maximum-intensity projections. Nuclei were stained with DAPI. Scale bar, 10 μm. Download FIG S4, JPG file, 2.1 MB. Copyright © 2018 Mittal et al. 2018 Mittal et al. This content is distributed under the terms of the Creative Commons Attribution 4.0 International license.

10.1128/mBio.01765-18.5 FIG S5 M. tuberculosis (Mtb) EsxG-EsxH dos not alter total cellular ESCRT levels. HeLa cells transfected with M. tuberculosis EsxG-EsxH were treated with or without LLOME for 15 min, and total cellular extracts were analyzed by Western blotting with the indicated antibodies. Download FIG S5, JPG file, 1.1 MB. Copyright © 2018 Mittal et al. 2018 Mittal et al. This content is distributed under the terms of the Creative Commons Attribution 4.0 International license.

### EsxG-EsxH responds to endomembrane damage.

**FIG 5:**
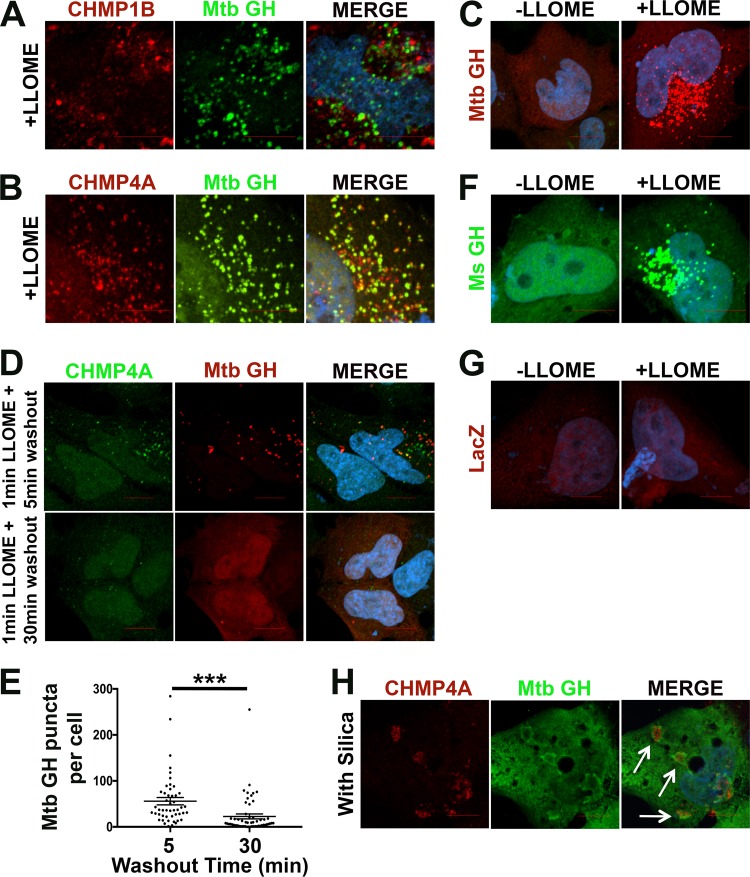
EsxG-EsxH relocalizes in response to membrane damage. HeLa cells transfected with M. tuberculosis (Mtb) EsxG-EsxH were treated with LLOME, and CHMP1B (A) or CHMP4A (B) (red) and EsxG-EsxH (green) was visualized by IF. (C) HeLa cells expressing Mtb EsxG-EsxH were treated with LLOME or the solvent control for 2.5 min. EsxG-EsxH is shown in red. (D) HeLa cells expressing Mtb EsxG-EsxH were treated with LLOME for 1.0 min and then incubated in excess LLOME-free medium for 5 to 30 min as indicated and stained for CHMP4A (green) and EsxG-EsxH (red). (E) Automated image analysis was used to quantify the number of CHMP4A punctae on 30 macrophages per sample from panel D. Data are means ± SEM from one representative experiment from at least two independent experiments. ***, *P* ≤ 0.001, Student's *t* test. (F and G) HeLa cells expressing Ms EsxG-EsxH (green) (F) or LacZ (vector control [red]) (G) were treated with LLOME or the solvent control. (H) U2OS cells transfected with Mtb EsxG-EsxH were treated with silica (SiO_2_) nanoparticles for 15 min and stained for CHMP4A (red) and EsxG-EsxH (green). White arrows indicate the silica nanoparticles. (A to H) Both EsxG-EsxH and LacZ were detected with anti-V5 antibody. Nuclei were stained with DAPI. Images are maximum-intensity projections. Scale bar 10 μm.

To better understand how EsxG-EsxH can modulate the host endomembrane damage response, we examined the behavior of these mycobacterial effectors themselves with regard to sterile endomembrane disruption. Unexpectedly, we found that EsxG-EsxH accumulated on numerous discrete punctae in cells treated with LLOME (Fig. 5). These structures colocalized partially with CHMP1B and CHMP4A (Fig. 5A and B), and they were apparent within minutes (Fig. 5C), similar to the rapid response previously reported for ESCRT-III (5). Brief exposure to LLOME has been shown to induce transient ESCRT-III recruitment to damaged endolysosomes (5). Similarly, we found that when cells were treated with LLOME for 1 min followed by removal of the compound, structures enriched for EsxG-EsxH formed quickly after LLOME addition and were largely resolved by 30 min after washout (Fig. 5D and E). The response of EsxG-EsxH to LLOME-induced damage was not a property specific to M. tuberculosis EsxG-EsxH, as the M. smegmatis EsxG-EsxH proteins exhibited the same behavior (Fig. 5F), whereas a control protein, LacZ, did not (Fig. 5G). The response of EsxG-EsxH was not specific to LLOME-induced damage, as damage induced by silica also resulted in the localization of EsxG-EsxH, along with CHMP4A, to the particles (Fig. 5H). Although both CHMP4A and EsxG-EsxH localized to silica, their staining patterns were quite distinct. EsxG-EsxH staining appeared even and continuous, adjacent to regions that had CHMP4A punctae. These data suggest that both M. smegmatis and M. tuberculosis EsxG-EsxH respond to sterile membrane damage, recognizing either the damaged membrane itself or recruited proteins.

### Relationship of HRS to EsxG-EsxH and the membrane damage response.

**FIG 6:**
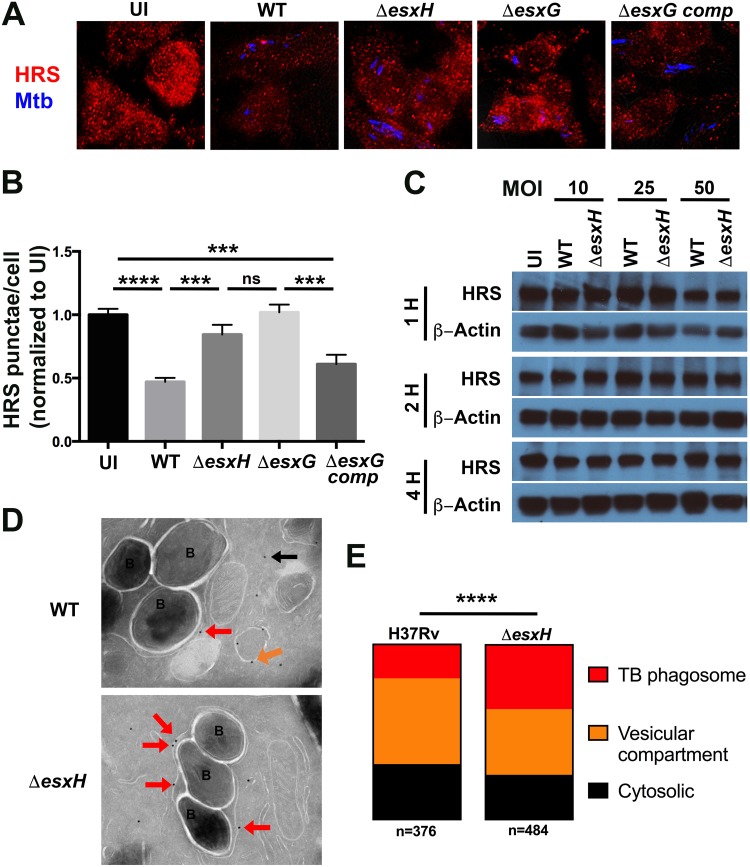
EsxG-EsxH alters HRS localization during infection. (A) BMDMs were uninfected (UI) or infected with H37Rv (WT), the Δ*esxH* or Δ*esxG* mutant, or the Δ*esxG* complemented strain for 3 h, and HRS was examined by IF. Mtb, M. tuberculosis. (B) The number of HRS punctae was quantified. Data are means ± SEM from three independent experiments. ****, *P* < 0.0001, and ***, *P* ≤ 0.0005, one-way ANOVA with Tukey’s multiple-comparison test. ns, not significant. (C) BMDMs were uninfected or infected with the WT or Δ*esxH* mutant for 1 to 4 h at a multiplicity of infection (MOI) of 10 to 50 as indicated. HRS and β-actin were examined by Western blotting. (D) Immunoelectron microscopy of BMDMs infected for 3 h with the WT and Δ*esxH* mutant. Red arrows indicate anti-HRS gold particles on M. tuberculosis phagosomes, while vesicular and cytosolic gold particles are indicated with orange and black arrows, respectively. Bacteria are labeled “B.” (E) The subcellular localization of anti-HRS gold particles was quantified from two independent experiments by an investigator blind to sample identity. At least 25 images with at least 174 bacilli per sample were analyzed. The number of anti-HRS gold particles in each sample is indicated (*n*). ****, *P* <0.0001, Fisher’s exact test.

We considered the possibility that EsxG-EsxH might be recruited to damaged endomembranes by interacting with ESCRT machinery, since we previously showed that EsxG-EsxH can bind the ESCRT-0 component HRS (35). In support of the idea that EsxG-EsxH acts on HRS during infection, macrophages infected with either WT or the Δ*pe5-ppe4* mutant bacteria exhibited reduced endosomal staining of HRS compared to uninfected macrophages (59), whereas no effect on HRS distribution was apparent in macrophages infected with the Δ*esxG* or Δ*esxH* mutant (Fig. 6A and B; see Fig. S6A and B in the supplemental material). There was no impact of infection or EsxG-EsxH on the distribution of another early endosomal protein, early endosomal antigen 1 (EEA1) (Fig. S6C and D). The decrease in HRS-positive structures seen during infection did not reflect a change in the amount of HRS protein, which was unaffected by infection or the presence of *esxH* (Fig. 6C). We also examined HRS localization by immunoelectron microscopy and confirmed that there was a significant difference in the protein’s distribution in macrophages infected with WT bacilli versus macrophages infected with the Δ*esxH* mutant (Fig. 6D and E). Notably, HRS associated significantly more with Δ*esxH* phagosomes than those containing WT bacilli. Thus, M. tuberculosis EsxG-EsxH alters the localization of HRS in infected macrophages and impairs recruitment of the protein to mycobacterial phagosomes.

10.1128/mBio.01765-18.6 FIG S6 HRS and EEA1 in the Δ*esxH* and Δ*pe5-ppe4* mutants. (A) IF images of HRS in BMDMs infected with the DsRed-expressing H37Rv or Δ*esxH* and Δ*pe5-ppe4* (Δ*ppe*) mutants for 3 h. Images are maximum-intensity projections. Scale bar, 10 μm. Mtb, M. tuberculosis. (B) Automated image analysis was used to quantify the number of HRS punctae in 30 macrophages per sample. (C and D) IF images of EEA1 in BMDMs that were uninfected or infected with indicated strains for 3 h. The number of EEA1 punctae was quantified 3 hpi. Data are means ± SEM from one representative experiment from at least three independent experiments. *, *P* ≤ 0.05, and ***, *P* ≤ 0.001, Student’s *t* test. ns, not significant. Download FIG S6, JPG file, 1.4 MB. Copyright © 2018 Mittal et al. 2018 Mittal et al. This content is distributed under the terms of the Creative Commons Attribution 4.0 International license.

**FIG 7:**
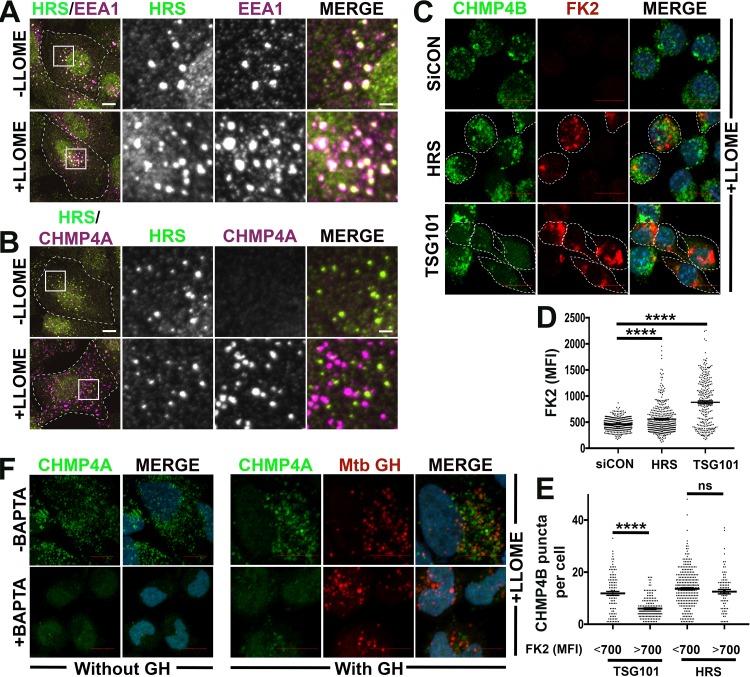
HRS is not required for ESCRT-III endomembrane damage response. (A and B) HRS does not assemble with ESCRT machinery on LLOME-disrupted endolysosomes. U20S cells were treated with LLOME or the solvent control for 10 min and then stained for HRS and EEA1 (A) or for HRS and CHMP4A (B). Boxed areas are magnified at right. The middle two columns show each indicated stain in grayscale; overlap of both stains in leftmost and rightmost columns appears white. Individual cells are outlined by white dashed lines; scale bars equal 10 μm (2 μm in magnified views). (C) RAW cells were treated with siRNA to deplete HRS or TSG101 for 2 days. Macrophages were then treated with LLOME for 15 min, and ubiquitin (FK2 antibody [red]) and CHMP4B (green) were examined. FK2-positive cells are outlined by white dashed lines. (D and E) Automated image analysis was used to quantify the mean fluorescent intensity (MFI) of FK2 (D) or the number of CHMP4B (E) punctae on 50 macrophages per sample. The number of CHMP4B punctae was compared in cells with FK2 MFI greater than and less than 700. Data are means ± SEM from one representative experiment from at least two independent experiments. ****, *P* ≤ 0.0001, Student’s *t* test. ns, not significant. (F) HeLa cells were transfected with M. tuberculosis (Mtb) EsxG-EsxH or vector control, preincubated for 1 h with BAPTA-AM, and then treated with LLOME for 15 min and stained for CHMP4A (green) and EsxG-EsxH (red). EsxG-EsxH was detected with anti-V5 antibody. Nuclei were stained with DAPI. Images are maximum-intensity projections. Scale bar, 10 μm.

During receptor trafficking, HRS serves as an adaptor to recruit downstream ESCRT complexes to receptors that have been ubiquitinated, thereby routing them for degradation (6). We accordingly asked whether HRS might also function as an adaptor to recruit ESCRT machinery to disrupted endomembranes. If so, the ability of EsxG-EsxH to bind HRS might explain both the localization of EsxG-EsxH to membrane damage, as well as the ability of EsxG-EsxH to antagonize damage-triggered ESCRT recruitment. However, when we examined HRS localization in LLOME-treated cells, we found that the protein remained associated with EEA1-marked early endosomes and did not localize to damaged endolysosomes that recruited CHMP4A (Fig. 7A and B). In addition, we previously showed that HRS preferentially binds M. tuberculosis EsxG-EsxH over M. smegmatis EsxG-EsxH, but here we found that EsxG-EsxH from both species responded similarly to LLOME-induced damage (Fig. 5C and F), suggesting that HRS is not what is driving their recruitment. To test whether HRS is required for the ESCRT-III response to endomembrane damage, we used previously validated small interfering RNAs (siRNAs) to deplete HRS (35, 42). As a positive control, we depleted cells of TSG101, a component of ESCRT-I, which we previously showed is required for damage-triggered ESCRT recruitment (5). Silencing of HRS and TSG101 resulted in the expected accumulation of ubiquitinated proteins on endosomes (Fig. 7C), consistent with their well-established role in receptor trafficking. As recently reported, the response of ESCRT-III to LLOME-induced damage was impaired in the absence of TSG101 (Fig. 7C) (5). In contrast, ESCRT-III responded indistinguishably to LLOME-induced damage in control cells and HRS-depleted cells, even though the HRS-silenced cells were defective in receptor trafficking (Fig. 7D). To quantify the effect of HRS and TSG101 silencing on the endomembrane damage response, we compared those cells within which the MFI of ubiquitinated proteins exceeded 700 (identifying those cells with impaired receptor trafficking) to those with a lower MFI. In the case of TSG101 silencing, there were fewer CHMP4B punctae in cells with impaired receptor trafficking (MFI of >700). In contrast, in those HRS-silenced cells that showed impaired receptor trafficking, there was no difference in the response of CHMP4B to LLOME (Fig. 7E). Thus, we conclude that HRS does not contribute significantly toward recruiting ESCRTs to disrupted endolysosomes.

Both EsxG-EsxH and ESCRT-III were recruited to LLOME-induced structures with similar kinetics (Fig. 5D and E), so we considered the possibility that EsxG-EsxH was recruited by ESCRT proteins other than HRS. To test whether the recruitment of EsxG-EsxH to sites of LLOME-induced damage depends upon the ESCRT-III response to such damage, we made use of the cell-permeable calcium chelator BAPTA-AM. As previously shown, when we pretreated cells with BAPTA-AM, CHMP4A no longer robustly responded to LLOME-induced damage (5). However, BAPTA-AM treatment had no impact on the response of EsxG-EsxH to such damage (Fig. 7F). Thus, EsxG-EsxH localizes to sites of endomembrane damage independently of ESCRT-III. Overall, we conclude that mycobacterial EsxG-EsxH antagonizes the recruitment of host ESCRT machinery to endomembrane damage in a manner that is unrelated to its interaction with HRS. Instead, EsxG-EsxH appears to recognize a feature of endomembrane damage independently of ESCRTs and therefore might modulate host membrane damage responses in a currently unappreciated way that includes inhibition of ESCRT recruitment.

## DISCUSSION

Specialized membrane-bound organelles are a defining feature of eukaryotic cells. In the endolysosomal network, foreign material and degradative enzymes are sequestered away from the cytosol. It was recently shown that the ESCRT machinery plays an important role in maintaining the integrity of this compartment, responding to and repairing minor damage caused by chemical and particulate insults. More extensive damage, including damage inflicted by intracellular pathogens, is detected by host galectins and cleared by autophagy. In the case of M. tuberculosis, phagosomal damage depends upon the ESX-1 secretion system and has been most definitively demonstrated after several days of infection. However, minor membrane perforation must happen within the first hours after infection, because macrophages quickly respond to bacterial products in the cytosol. Here, we show that ESCRT-III proteins are recruited to M. tuberculosis phagosomes during these first hours of infection. WT bacilli recruit ESCRT-III much more extensively than mutants lacking *esxA*, consistent with the idea that ESCRT-III is responding to endomembrane damage. Although we observed much less recruitment of ESCRT-III to the *esxA* mutant than WT bacilli, we did still see some recruitment, suggesting there is a low level of *ESX-1*-independent phagosomal damage as well. Interestingly, after M. tuberculosis infection, the number of ESCRT-III punctae increased throughout the cell, suggesting that M. tuberculosis damages subcellular compartments beyond the phagosome. We speculate that the damage is caused by ESX-1 effector(s) and PDIM trafficking through the endolysosomal system, as has been shown for a number of other M. tuberculosis surface proteins and lipids (60, 61).

The idea that ESCRT responds to endosomal perturbation from microorganisms fits well with our previous studies and those of others showing that ESCRT plays an evolutionarily conserved role controlling intracellular microbes. RNA interference (RNAi) screens and follow-up studies demonstrated that ESCRT is important in restricting the growth of a variety of microbes, including rapidly growing mycobacteria such as Mycobacterium fortuitum and Mycobacterium smegmatis, as well as slow growers, including M. bovis BCG and M. tuberculosis (34–36). We propose that even though M. smegmatis, M. fortuitum, and M. bovis BCG do not have dedicated systems to breach the phagosomal membrane, they still cause minor perturbations, which without ESCRT-mediated repair, would compromise compartmental integrity and, in particular, compartmental pH. This could explain why ESCRT silencing impairs lysosomal trafficking and phagosomal acidification not only for M. tuberculosis, but also for these nonpathogenic mycobacteria. Moreover, consistent with the idea that ESCRT maintains phagosomal integrity, RNAi screens in Listeria demonstrated that mutants lacking the cytolysin LLO, which normally fail to enter the host cytosol, are able to do so in cells lacking ESCRT (62). We also found previously that ESCRT silencing impairs antigen processing and MHC-II antigen presentation (42), and defective endomembrane integrity might contribute to these findings. Interestingly, in gamma interferon (IFN-γ)-activated macrophages, there is no effect of ESCRT silencing on mycobacterial phagolysosomal trafficking or host control of infection (42). We speculate that because IFN-γ induces autophagy (43), which can also resolve endomembrane damage, activated macrophages are less dependent upon ESCRT-mediated repair. In addition, IFN-γ activates a Rab20-dependent pathway that promotes the formation of spacious vacuoles and thereby preserves phagosome integrity (63). Activated macrophages may thus minimize their need for ESCRT-promoted repair. Overall, we favor the idea that a major contribution of ESCRT to control of intracellular bacteria is related to its function in endomembrane repair, which is important for maintaining endolysosomal competence, restricting cytosolic access of microbes, and dampening inflammatory signaling. We cannot rule out the possibility that ESCRT is important in phagosome maturation based upon its role in receptor trafficking, but it is less clear how this would work. In addition, ESCRT participates in diverse aspects of cell biology, including plasma membrane repair and exosome formation, which may all be significant, depending on the nature of the infection.

**FIG 8:**
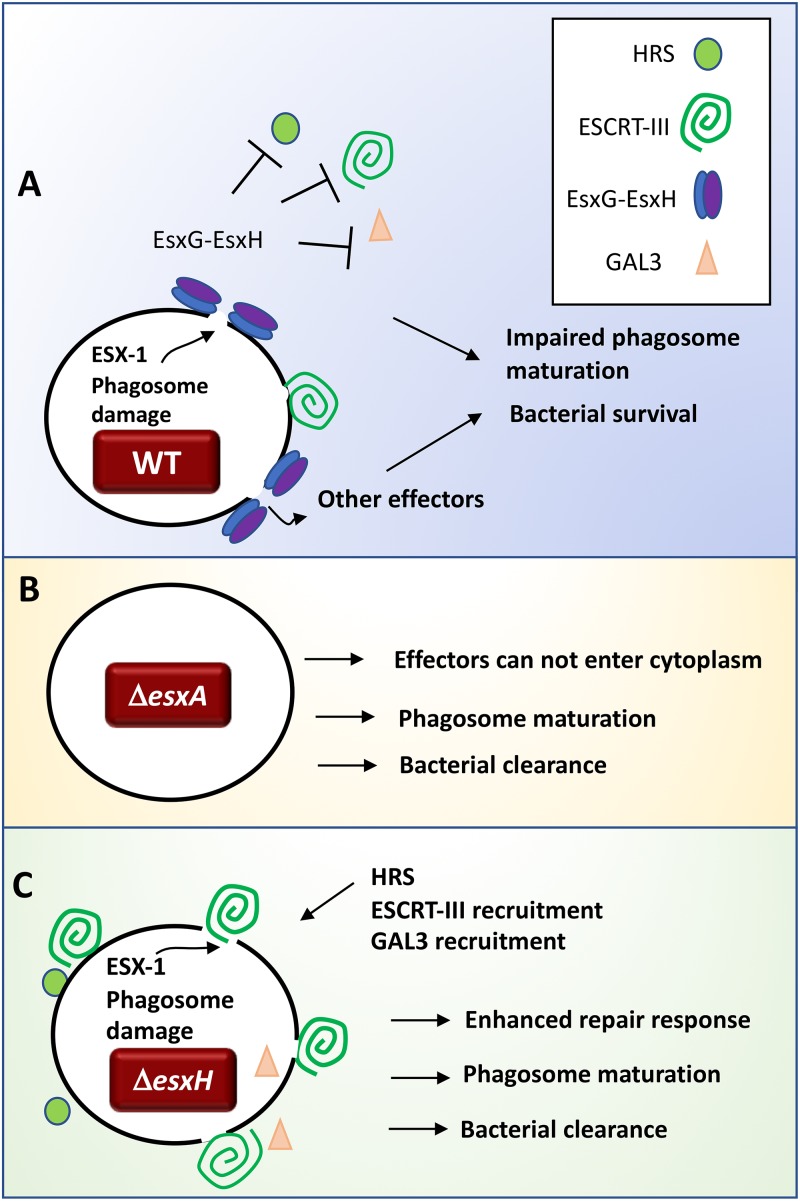
Model depicting how the presence of ESCRT at the M. tuberculosis phagosome is determined by ESX-1 and ESX-3. (A) In WT bacilli, ESX-1 effectors generate phagosomal damage. EsxG-EsxH antagonizes recruitment of HRS, ESCRT-III, and GAL3 to the phagosome. EsxG-EsxH alters HRS localization during infection, which might impair ESCRT-III recruitment in the context of receptor trafficking, but is unlikely to account for ESCRT-III inhibition in response to endomembrane damage. (B) During infection with a Δ*esxA* mutant, there is reduced phagosome damage. Without phagosomal perforation, M. tuberculosis is impaired in its ability to manipulate cellular trafficking and immune responses, and therefore, the bacilli are cleared. (C) Infection with Δ*esxH* mutants results in enhanced recruitment of HRS, ESCRT-III, and GAL3 to bacilli, which interferes with the bacterial virulence program.

The idea that ESCRT plays an important role in host control of infection is underscored by our findings that M. tuberculosis EsxG-EsxH impairs ESCRT. Our previous studies showed that EsxG-EsxH plays a critical role in M. tuberculosis virulence: mutants lacking EsxH are highly attenuated in mice and impaired in macrophages, phenotypes that are not explained by defective iron uptake and which are not complemented by the EsxG-EsxH homologues from M. smegmatis (41). We propose that T7SS effectors and ESCRT participate in a series of measures and countermeasures that control of phagosome integrity (Fig. 8). In the case of WT M. tuberculosis, the ESX-1 system, along with PDIM, damages the phagosomal membrane. At the same time, EsxG-EsxH prevents recruitment of ESCRT to sites of damage, ensuring sustained phagosomal perforation and contributing to impaired lysosomal maturation (Fig. 8A). In the case of the Δ*esxA* mutant, membrane damage is minimal, and there is correspondingly little ESCRT recruitment. Since the Δ*esxA* mutant is unable to efficiently deliver effectors to the cytosol, it is cleared (Fig. 8B). During infection with the Δ*esxH* mutant, damage occurs, and in the absence of EsxG-EsxH, ESCRT is more robustly recruited than in WT infections (Fig. 8C).

Although ESCRT-III is recruited to the Δ*esxH* mutant, it is not clear to what degree it can repair pathogen-induced damage in this context. Since we observed discordant results in the recruitment of GAL3 and ubiquitin—both markers of phagosomal damage—it is difficult to infer the fate of the damaged membrane. Although GAL3 is recruited to damaged phagosomes by binding endosomal glycoproteins that become exposed to the cytosol, it has also been reported to interact with ESCRT-tethering proteins ALIX and TSG101 (64–66), so GAL3 may be recruited or retained independently of damage. Ubiquitin deposition is also an indirect measure of phagosomal damage, and, furthermore, is influenced by endocytic cargo sorting. Moreover, since GAL3 is reported to inhibit parkin-mediated ubiquitination (67), the two markers may not be independent. Despite uncertainty about whether ESCRT-III repairs the damage during infection with the Δ*esxH* mutant, it is clear that without EsxG-EsxH there is a more robust ESCRT response at the mycobacterium-containing phagosomal membrane, and the pathogen no longer successfully orchestrates its virulence program. Given the importance of phagosome integrity for the host and pathogen, both in terms of microbial control and also inflammatory responses, it seems likely that other pathogens, including viruses, engage ESCRT over its role in membrane repair.

How might EsxG-EsxH antagonize ESCRT? One possibility is that the difference in the ESCRT response to infection with the Δ*esxH* mutant is related to intrinsic differences in the bacteria. For example, EsxG-EsxH might inhibit the membranolytic activity of M. tuberculosis. In this case, ESCRT would be more robustly recruited to the Δ*esxH* mutant because the mutant generates more membrane damage than WT bacilli. However, at least *in vitro*, there is no cross-regulation of the ESX-1 and ESX-3 systems in terms of effector secretion (41, 68). It is possible that after they are secreted from the bacilli, ESX-1 and ESX-3 effectors have opposing activities at the phagosome. However, we found that EsxG-EsxH can also impair ESCRT-III recruitment in the context of sterile damage, suggesting that EsxG-EsxH operates independently of other bacterial factors. We favor the idea that EsxG-EsxH antagonizes ESCRT by virtue of interacting with either host membranes or proteins. Although EsxG-EsxH can bind HRS (35) and alter HRS localization during infection (Fig. 6), our data suggest that HRS is not the relevant ESCRT adaptor in the context of the endomembrane damage response (Fig. 7). We think EsxG-EsxH is likely to have another activity that allows it to inhibit ESCRT-III recruitment in response to endomembrane damage during infection. Since EsxG-EsxH relocalizes in response to membrane damage, another possibility is that EsxG-EsxH competes with ESCRT recognition or binding to sites of damage. However, since both the M. smegmatis and M. tuberculosis EsxG-EsxH proteins respond to damaged membranes, but only the M. tuberculosis proteins promote virulence and impair lysosomal trafficking (35, 41, 42), M. tuberculosis EsxG-EsxH must have additional activity. So far, the only M. tuberculosis-specific function identified for EsxG-EsxH is its ability to interact with HRS (35), but our findings here suggest an additional role or roles. Further work is required to elucidate exactly how EsxG-EsxH impairs ESCRT and promotes disease. Given the critical importance of this effector in virulence and immune responses (42, 69, 70), understanding how it perturbs host cellular function is central to understanding M. tuberculosis pathogenesis.

## MATERIALS AND METHODS

### Bacterial strains and growth conditions.

The following M. tuberculosis strains were used in this study: H37Rv (WT), the Δ*esxA* mutant (32), the Δ*esxG* mutant (mc^2^7789), the Δ*esxG* complement (mc^2^7789 with pYUB1335), the Δ*esxH* mutant (mc^2^7846), the Δ*pe4-ppe5* (Δ*ppe*) mutant (mc^2^7848), the Δ*esxH* complement (mc^2^7846 containing pJP130), mc^2^6206 (Δ*leuCD* Δ*panCD*), and mc^2^6230 (Δ*RD1* Δ*panCD*). The Δ*esxA* mutant, mc^2^6206, and mc^2^6230 were provided by William Jacobs, Jr. (Albert Einstein College of Medicine), and have also been previously characterized by our laboratory (32, 52, 53, 71). The Δ*esxG*, Δ*esxH*, and Δ*pe4-ppe5* (Δ*ppe*) mutants and complementing plasmids were previously described in detail (41, 42). Previously, we showed that mc^2^6230 exhibits reduced LC3 trafficking compared to mc^2^6206, consistent with the reduction in xenophagy described for *ESX-1* mutants (71). Strains were grown at 37°C in 7H9 medium (Middlebrook 7H9 broth; Difco) supplemented with 0.05% Tween 80 (Sigma), BBL Middlebrook OADC (oleic acid-albumin-dextrose-catalase) enrichment, and 0.2% glycerol (Sigma). DsRed, pYUB1335, and pJP130 plasmids were selected with 25 μl/ml kanamycin. mCherry-expressing plasmids were selected with 50 μl/ml hygromycin. Medium was supplemented with d-pantothenic acid (24 μg/ml) for strains lacking *panCD* and l-leucine (50 μg/ml) for strains lacking *leuCD*.

### Tissue culture conditions.

Murine hematopoietic stem cells were isolated from the tibias and femurs of 6- to 15-week-old C57BL/6 mice. For differentiation into BMDMs, DMEM (Dulbecco’s modified Eagle’s medium [Gibco]) was supplemented with 10% fetal bovine serum (FBS) and 20% L929-conditioned medium for 7 days. The concentration of L929-conditioned medium was reduced to 10% before infections. HeLa cells obtained from Michael S. Diamond (Washington University School of Medicine) were grown in a mixture of DMEM, 2 mM l-glutamine, and 10% heat-inactivated fetal bovine serum (hiFBS [Invitrogen]). Penicillin/streptomycin (Gibco) was added for passaging of cells and excluded during infection of BMDMs. U2OS human osteosarcoma cells and RAW264.7 mouse macrophages originally from the American Type Culture Collection (ATCC [Manassas, VA]) were grown in DMEM supplemented with 10% FBS. Plasmids were transfected into HeLa cells with Effectene (Qiagen). Cells were grown at 37°C in a 5% CO_2_ atmosphere.

### Plasmid construction.

For EsxG-EsxH expression in mammalian cells, M. tuberculosis EsxG and EsxH were amplified by PCR using oligonucleotides that added 5′ HindIII and 3′ BamHI sites (for EsxG) and 5′ BamHI and 3′ V5 tag-XbaI (for EsxH). Both EsxG and EsxH-V5 were cloned into pcDNA3.1(+). A self-cleaving viral 2A peptide sequence (EGRGSLLTCGDVEENPGP) with flanking BamHI overhangs was generated by annealing of complementary oligonucleotides and cloned into the BamHI restriction site between EsxG and EsxH to generate EsxG-T2A-EsxH-V5 (pET018) and confirmed by sequencing. To generate the M. smegmatis EsxG-T2A-EsxH-V5 plasmid (pEM01), Genewiz synthesized DNA that was identical to the insert of pET018, except that the M. tuberculosis EsxG and EsxH sequences were replaced with M. smegmatis EsxG and EsxH, and then the insert was cloned into the HindIII and XbaI restriction sites of pET018.

### RNAi-mediated silencing.

siRNAs for TSG101 (SMARTpool-ON-TARGET*plus*, L-049922-01) and HRS (ON-TARGET*plus,* J-055516-09) were transfected using Hiperfect (Qiagen). RNAi-mediating silencing of TSG101 and HRS has been reported previously (35). Silencing was performed with 50 nM siRNA for 2 days before LLOME treatment. ON-TARGET*plus* nontargeting siRNA 1 (D-001210-01) was used as a control.

### Microscopic analysis of infected cells.

For immunofluorescence (IF) microscopy, BMDMs (1 × 10^5^) were seeded on 12-mm coverslips in 24-well plates 1 day prior to infection. Macrophages were infected with DsRed-expressing, mCherry-expressing, or PKH-labeled M. tuberculosis at an MOI of ∼5. After 3 h, macrophages were washed with phosphate-buffered saline (PBS) and fixed with 1% paraformaldehyde–PBS overnight followed by permeabilization in 0.1% vol/vol Triton X-100 (Sigma-Aldrich) in PBS for 10 min at room temperature and blocked for 45 min in 2% bovine serum albumin (BSA) in PBS prior to immunostaining with the primary and secondary antibodies listed below. Samples were stained with DAPI (4′,6-diamidino-2-phenylindole) and mounted in Prolong Diamond antifade (Molecular Probes, Life Technologies). Images were captured using a Nikon Eclipse Ti confocal microscope (Nikon Instruments, Inc.) equipped with a 60× apochromat oil objective lens. Image acquisition and analysis were done using NIS-Elements version 4.40. Briefly, a region of interest (ROI) was drawn in close apposition around each bacterium or cell (depending upon the experiment), and the fluorescence intensity was measured using the ROI tool. The numbers of punctae were measured with a spot detector.

In the case of HRS and EEA1 IF in BMDMs, cells were seeded in 8-well chamber slides at 1 × 10^5^ per well. The next day, they were infected and fixed as described above. Cells were immunostained with HRS (72) and EEA1 (rabbit antibody ab2900; Abcam) in 0.1% saponin–2% BSA in PBS. In those experiments, M. tuberculosis was visualized based upon its autofluorescence using the DAPI filter. The images were acquired using Nikon Eclipse TiE/B automated fluorescence microscope with 60×; Plan-apochromat, NA 1.4 oil immersion objective, Photometric Cool SNAP HQ2 monochrome digital camera and appropriate filter sets for DAPI and TexasRed channel. The 60× z-stack images were acquired, deconvoluted, and analyzed using Nikon Imaging Software-Elements Advanced Research (NIS-Elements AR, v3.2). The cellular distribution of HRS and EEA1 was quantified by thresholding images to select punctae. The number of punctae was divided by the cell number for each field to arrive at the number of punctae per cell. At least three fields per sample with an average number of 20 cells per field were acquired for image analysis. The data were normalized to uninfected samples.

### Endolysosomal membrane damage.

For continuous LLOME treatments, cells seeded on 12-mm coverslips were exposed to 1 mM LLOME in complete growth medium for 10 to 15 min, unless otherwise indicated, and then fixed in 4% paraformaldehyde in PBS for 20 min at room temperature and immunostained as described above. For pulse-chase experiments, coverslips were immersed in medium containing LLOME for 1 min, rinsed in drug-free medium, and then incubated in excess drug-free medium before processing for immunofluorescence. Where indicated, cells were transfected for 30 to 40 h before LLOME treatment with 400 ng of pET018, pEM01 (described above), or empty vector (pJP115/pcDNA/GW40/*lacZ* [Invitrogen]). For experiments assessing the effect of calcium on cellular responses to endolysosomal damage, cells were incubated in medium containing 25 μM BAPTA-AM for 1 h and then switched to medium containing 25 μM BAPTA-AM together with LLOME for an additional 15 min. Images were acquired and analyzed as described above.

### Silica crystal nanoparticle uptake.

Silica nanoparticles (no. tlrl-sio; InvivoGen) were suspended in ultrapure water according to the manufacturer’s instructions and diluted in complete growth medium to 100 μg/ml. The resulting suspension was added to subconfluent cells on glass coverslips, and cells were incubated for 15 min before being fixed and prepared for immunofluorescence.

### Time-lapse recording for SRB assay.

HeLa cells were seeded in a four-chamber, no. 1.5 glass-bottom dish (Cellvis) and cultured overnight before being transfected for 36 h with pET018. Transfected cells were next incubated for 6 h in complete growth medium supplemented with the red fluorescent dye sulforhodamine B (SRB [S1307]; Molecular Probes) at 200 ng/µl and then extensively washed and chased for 6 h in growth medium lacking SRB to allow the dye to accumulate in late endosomes and lysosomes. Before imaging, each dish was inscribed with three fiduciary marks to facilitate alignment on the microscope stage (described below). Medium was then replaced with warmed imaging buffer (20 mM HEPES [pH 7.4] at room temperature, 140 mM NaCl, 2.5 mM KCl, 1.8 mM CaCl_2_, 1 mM MgCl_2_, 10 mM d-glucose, and 5% vol/vol FBS), and the dish was equilibrated for at least 20 min in an INU series stage-top incubator (Tokai Hit) at 37°C. Additional imaging buffer, intended for preparing drug solutions, was simultaneously warmed in a separate vessel placed within the stage-top incubator together with the cells. A grid of individual fields, covering a total area of approximately 1 mm^2^, was imaged at 5-min intervals for a total of 30 min. After the initial time point, acquisition was briefly paused and buffer was replaced with fresh imaging solution containing 1 mM LLOME. Recordings were acquired on a spinning disk confocal platform consisting of a Ti-E inverted microscope (Nikon Instruments) and a CSU-X1 variable speed scanner (Yokogawa), using a Nikon 60× 1.40 NA CFI Plan apochromat lambda oil immersion objective, a 561-nm laser, a red emission single-band bandpass filter (605 ± 35 nm), and a Zyla 4.2-megapixel sCMOS camera (Andor), using a 16-bit dual amplifier without binning. Axial drift was compensated using the Nikon Perfect Focus System (PFS). At the end of each recording, cells were fixed for 15 min at room temperature in 4% wt/vol paraformaldehyde in PBS and immunostained as described above. Dishes were next aligned on the microscope stage using the inscribed fiduciary marks, and the immunostained cells were imaged by optical sectioning on the same spinning disk confocal platform described above, using 488-nm and 640-nm lasers and green emission (525 ± 18 nm) and far-red emission (700 ± 37.5 nm) single-band bandpass filters. Maximum-intensity projections of each immunostain were then manually aligned to the time-lapse recordings of SRB fluorescence in Nikon NIS-Elements software. Image analysis was performed as described above.

### Antibodies and other materials.

LLOME (no. L7393; Sigma-Aldrich) and BAPTA-AM (no. 15551; Cayman Chemical) were prepared in dimethyl sulfoxide (DMSO) and stored at −80°C in single-use aliquots. The PKH26 red fluorescent cell linker midi kit (no. MIDI26-1KT), d-pantothenic acid (no. 21210), and radioimmunprecipitation assay (RIPA) buffer were purchased from Sigma-Aldrich. Silica nanoparticles (no. tlrl-sio) and SRB (no. S1307) were purchased from InvivoGen and Molecular Probes, respectively. siRNAs for TSG101 (SMARTpool-ON-TARGET*plus,* L-049922-01), HRS (ON-TARGET*plus,* J-055516-09), and ON-TARGETplus nontargeting siRNA 1 (D-001210-01) were obtained from Dharmacon. l-Leucine (no. 194694) was purchased from MP Biomedicals.

The following antibodies were used: ALIX, rabbit, 12422-1-AP (ProteinTech); CHMP1A, mouse, sc-271617 (Santa Cruz); CHMP1B, rabbit, 14639-1-AP (ProteinTech), and mouse, sc-514013 (Santa Cruz); CHMP4A, rabbit (73); CHMP4B, rabbit, clone 485 (A. Shiels, Washington University School of Medicine); IST1, rabbit, 19842-1-AP (ProteinTech); EEA1, rabbit, 2411 (Cell Signaling Technology) (for Fig. 7A and B), and rabbit, ab2900 (Abcam) (for Fig. 6AB and Fig. S6A and B); LAMP1, rabbit, NB120-19294 (Novus); HRS, mouse (Andrew Bean, The University of Texas Health Science Center at Houston Medical School, Houston), and mouse, ALX-804-382 (Enzo Life Sciences); β-actin, mouse, BA3R, MA5-15739 (Invitrogen); V5-tag, mouse, 377500 (Life Technologies); GAL3, rabbit, sc-20157 (Santa Cruz); polyubiquitin, mouse, clone FK2, 04-263 (Millipore Sigma); anti-EsxG-EsxH, clone 2D1.C3.H5 IgG2b (this study); goat anti-mouse IgG-horseradish peroxidase (HRP), 31430, (Pierce); goat anti-rabbit IgG-HRP, 32260 (Invitrogen); Alexa Fluor 488-conjugated goat anti-mouse IgG, A11029 (Invitrogen); Alexa Fluor 594-conjugated goat anti-mouse IgG, A11032 (Invitrogen); Alexa Fluor 488-conjugated goat anti-rabbit IgG, A11034 (Invitrogen); Alexa Fluor 594-conjugated goat anti-rabbit IgG, A11037 (Invitrogen); and Alexa Fluor 647-conjugated goat anti-rabbit IgG, A21245 (Invitrogen).

To generate monoclonal antibodies against M. tuberculosis EsxG-EsxH, 250 µg of recombinant His-tagged EsxG-EsxH was produced in Escherichia coli as previously described (35) and injected into mice using TiterMax Gold adjuvant, followed by boosts with incomplete Freund’s adjuvant containing 125 µg purified EsxG-EsxH. Fusion experiments were performed according to standard procedures. Supernatants from hybridoma clones were screened by indirect enzyme-linked immunosorbent assay (ELISA) and IF microscopy. Single clones were isolated by repeated limiting dilution cloning. Established hybridomas were cultivated, and MAbs were purified from the conditioned medium by using NAb protein A/G spin columns (ThermoFisher, catalog no. 89958) per the manufacturer's instructions for gravity-flow purification.

### Electron microscopy.

BMDMs (1 × 10^6^/well) were seeded in a 6-well plate 1 day before infection. Macrophages were infected with H37Rv (WT) or the Δ*esxH* mutant at an MOI of ∼10. After 3 h, macrophages were washed and fixed in 1% paraformaldehyde–0.04% glutaraldehyde (Polysciences, Inc., Warrington, PA) in 100 mM PIPES [piperazine-*N*,*N*′-bis(2-ethanesulfonic acid)] buffer overnight, followed by embedding in 10% gelatin overnight with 2.3 M sucrose–20% polyvinylpyrrolidone in PIPES at 4°C. Samples were frozen in liquid nitrogen and sectioned with a cryo-ultramicrotome. Sections were probed with primary HRS antibody (from A. Bean) followed by colloidal conjugated secondary antibody (Jackson ImmunoResearch Laboratories), stained with uranyl acetate/methylcellulose, and analyzed by transmission electron microscopy.

### Protein extracts and Western blotting.

BMDMs (1 × 10^6^/well) were seeded in a 6-well plate 1 day before infection. BMDMs were infected with H37Rv or the Δ*esxH* mutant for 1, 2, and 4 hpi at different MOI (10, 25, and 50). Cells were removed from culture dishes, washed in PBS, and lysed in RIPA buffer (150 mM NaCl, 1.0% IGEPAL CA-630, 0.5% sodium deoxycholate, 0.1% SDS, 50 mM Tris [pH 8.0]) and subjected to Western blotting. Blots were probed with indicated antibodies. HeLa cells transfected with indicated constructs were cultured for 40 h and subsequently treated with or without 1 mM LLOME for 15 min. After LLOME treatment, cells were washed with PBS and lysed in RIPA buffer and then subjected to Western blotting. The blots were probed with the indicated antibodies.

### Mice.

The Washington University School of Medicine Institutional Animal Care and Use Committee approved all work with mice. Euthanasia was performed prior to bone marrow harvest in accordance with the 2013 *AVMA Guidelines for the Euthanasia of Animals* (https://www.avma.org/KB/Policies/Documents/euthanasia.pdf).

### Image analysis and reproduction.

For all microscopy experiments, contrast was not altered before image analysis. In cases where the contrast was enhanced for reproduced images, the adjustment was the same for all samples.

### Statistical analysis.

The data shown are representative of 2 or more independent experiments. In all figures, error bars indicate the mean ± standard error of the mean (SEM). The unpaired, two-tailed Student's *t* test, one-way analysis of variance (ANOVA) with Tukey’s multiple-comparison test, or Fisher's exact test was used to assess the statistical significance of the comparison of experimental groups using GraphPad Prism software.
