# Supplementary figures and images for: Mycobacterium tuberculosis Type VII Secretion System Effectors Differentially Impact the ESCRT Endomembrane Damage Response
Source: mBio. 2018 Nov 27;9(6):e01765-18. doi: 10.1128/mBio.01765-18 (PMC6282207; doi:10.1128/mBio.01765-18)

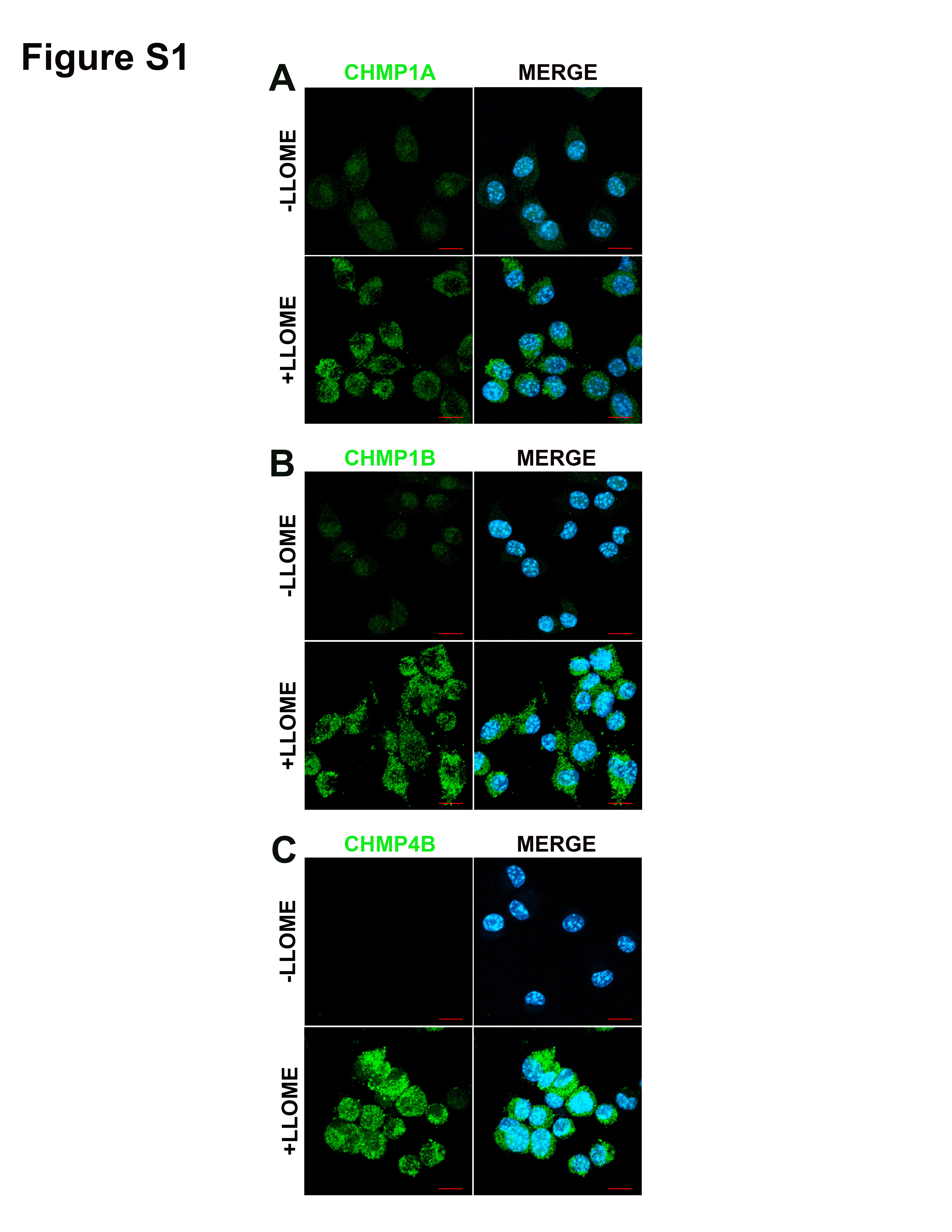

Supplement: FIG S1 [file mbo006184190sf1.jpg]

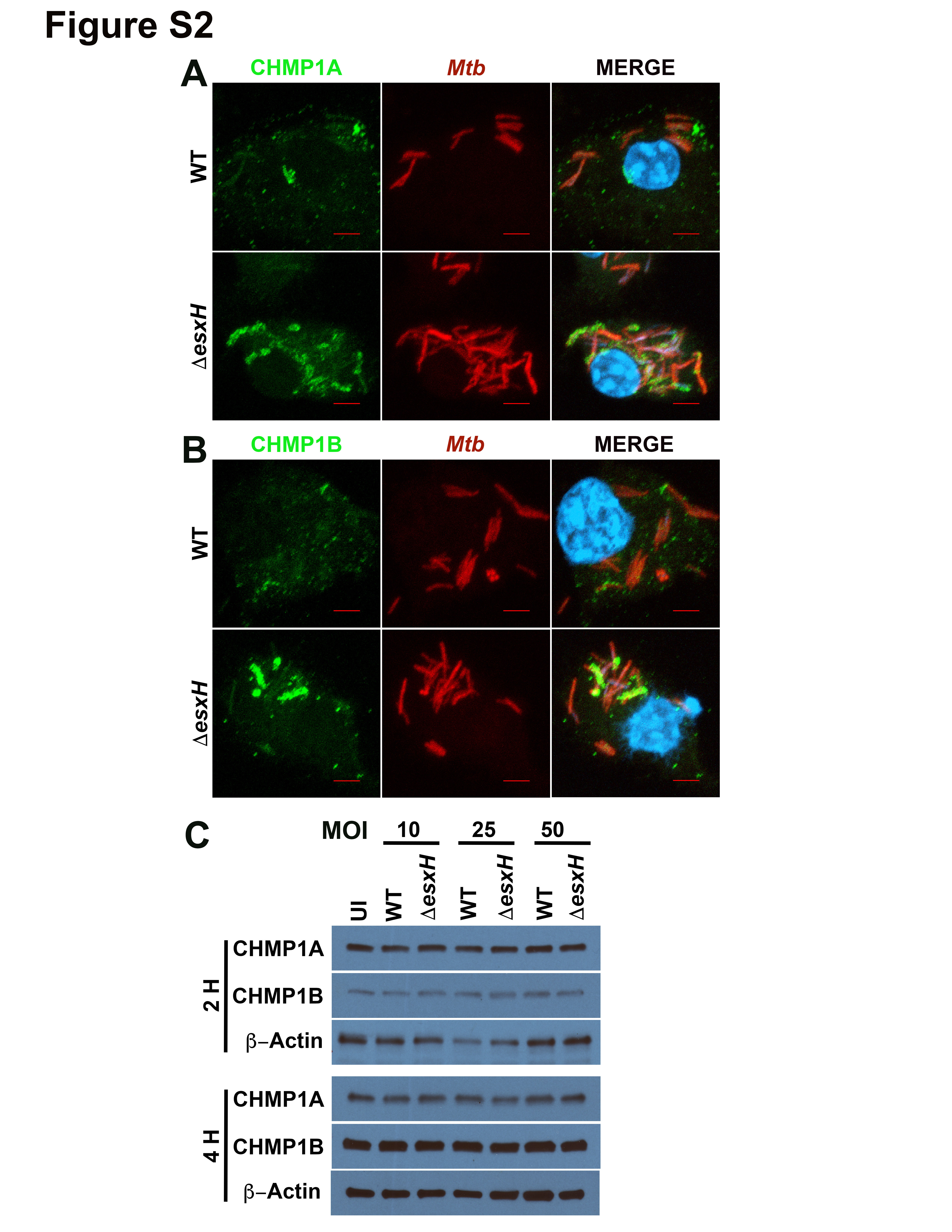

Supplement: FIG S2 [file mbo006184190sf2.jpg]

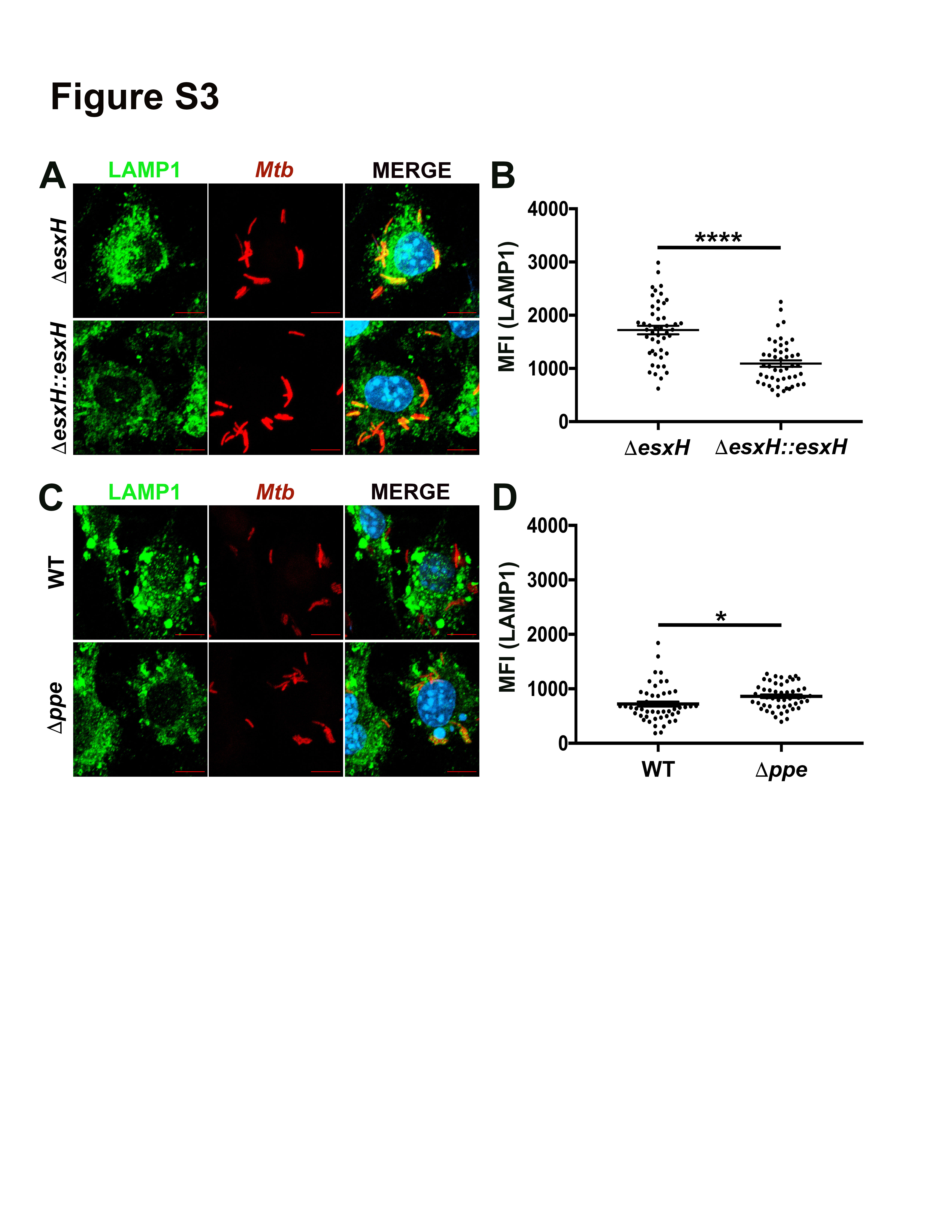

Supplement: FIG S3 [file mbo006184190sf3.jpg]

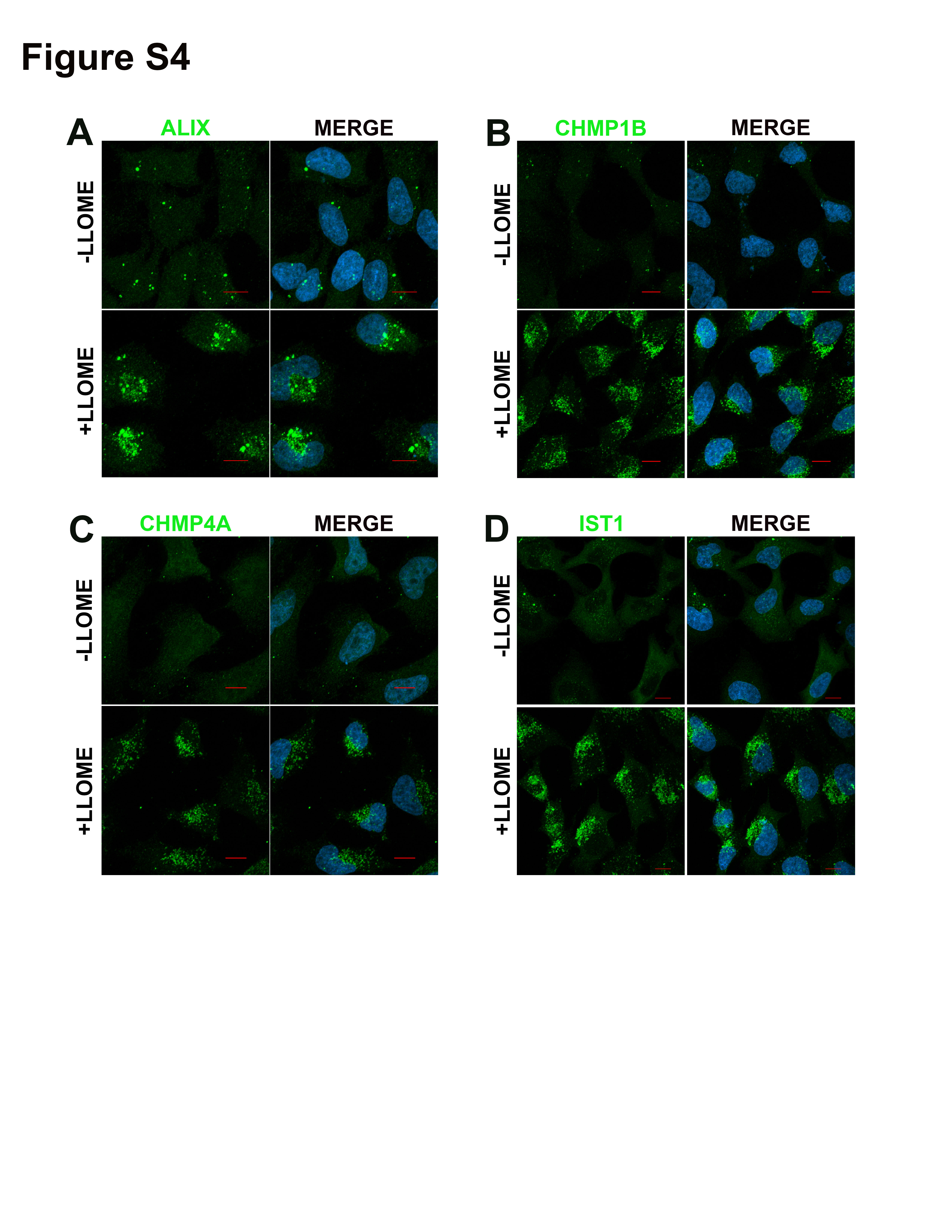

Supplement: FIG S4 [file mbo006184190sf4.jpg]

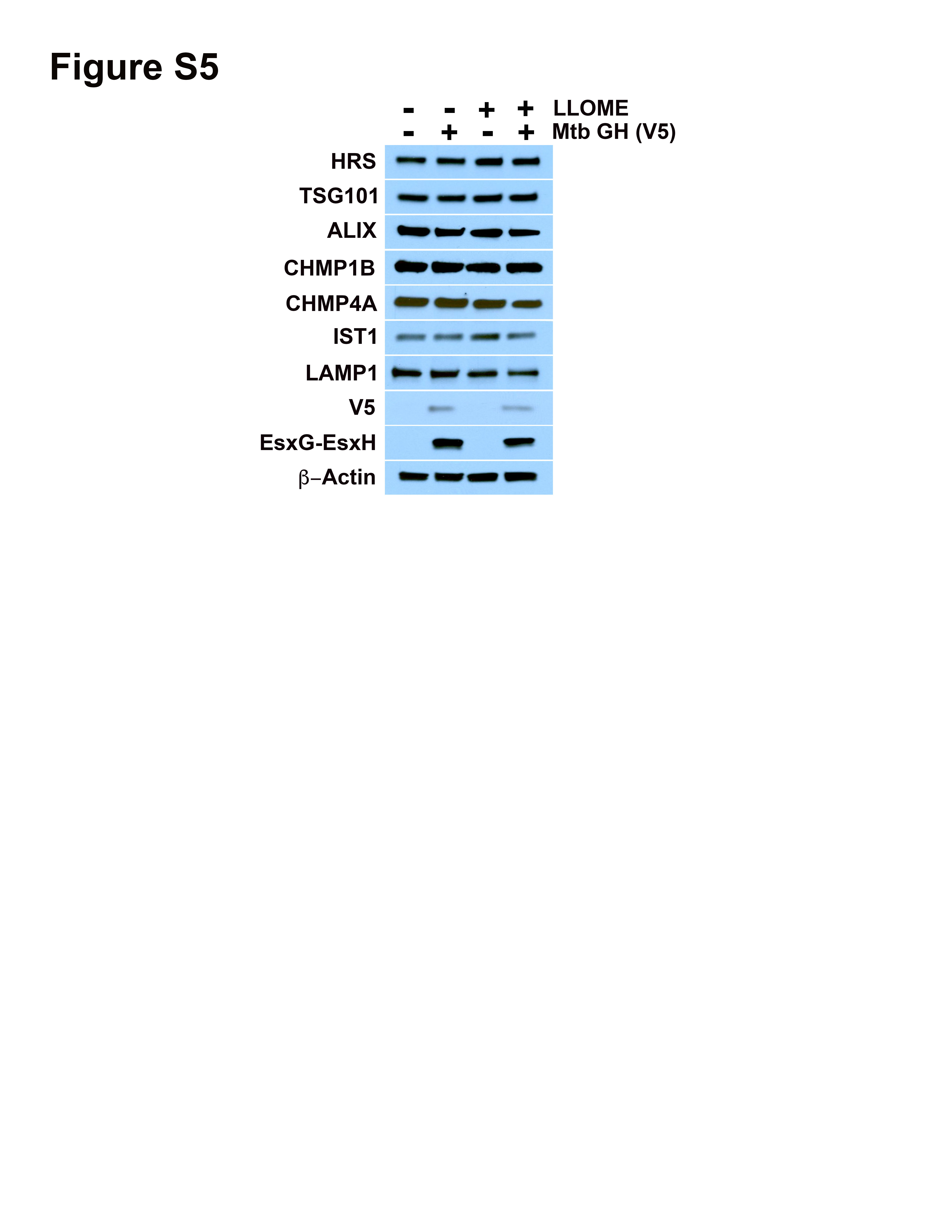

Supplement: FIG S5 [file mbo006184190sf5.jpg]

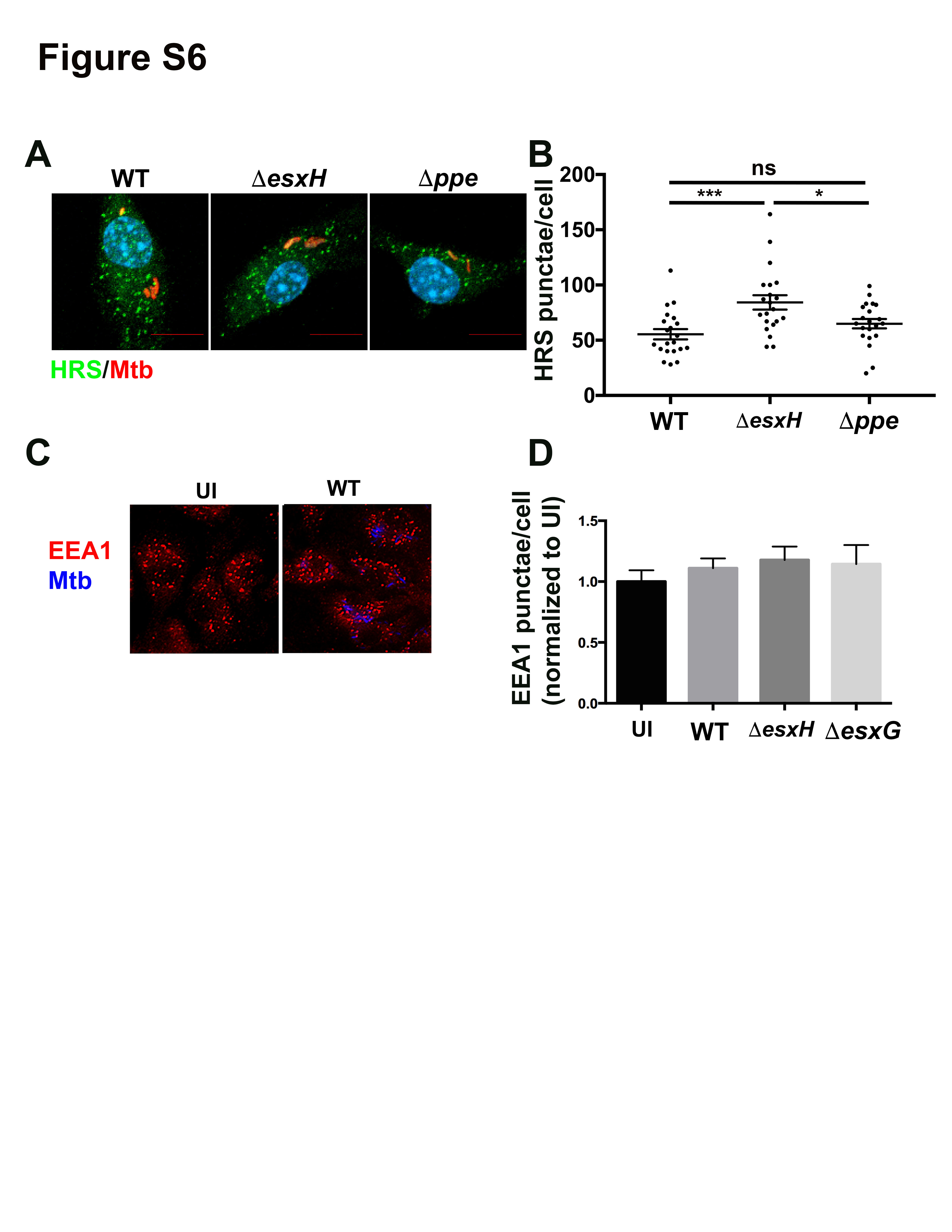

Supplement: FIG S6 [file mbo006184190sf6.jpg]
